# HRS plays an important role for TLR7 signaling to orchestrate inflammation and innate immunity upon EV71 infection

**DOI:** 10.1371/journal.ppat.1006585

**Published:** 2017-08-30

**Authors:** Zhen Luo, Maolin Ge, Junbo Chen, Qibin Geng, Mingfu Tian, Zhi Qiao, Lan Bai, Qi Zhang, Chengliang Zhu, Ying Xiong, Kailang Wu, Fang Liu, Yingle Liu, Jianguo Wu

**Affiliations:** 1 State Key Laboratory of Virology, College of Life Sciences, Wuhan University, Wuhan, China; 2 Institute of Medical Microbiology, Jinan University, Guangzhou, China; University of Wisconsin, UNITED STATES

## Abstract

Enterovirus 71 (EV71) is an RNA virus that causes hand-foot-mouth disease (HFMD), and even fatal encephalitis in children. Although EV71 pathogenesis remains largely obscure, host immune responses may play important roles in the development of diseases. Recognition of pathogens mediated by Toll-like receptors (TLRs) induces host immune and inflammatory responses. Intracellular TLRs must traffic from the endoplasmic reticulum (ER) to the endolysosomal network from where they initiate complete signaling, leading to inflammatory response. This study reveals a novel mechanism underlying the regulation of TLR7 signaling during EV71 infection. Initially, we show that multiple cytokines are differentially expressed during viral infection and demonstrate that EV71 infection induces the production of proinflammatory cytokines through regulating TLR7-mediated p38 MAPK, and NF-κB signaling pathways. Further studies reveal that the expression of the endosome-associated protein hepatocyte growth factor-regulated tyrosine kinase substrate (HRS) is upregulated and highly correlated with the expression of TLR7 in EV71 infected patients, mice, and cultured cells. Virus-induced HRS subsequently enhances TLR7 complex formation in early- and late-endosome by interacting with TLR7 and TAB1. Moreover, HRS is involved in the regulation of the TLR7/NF-κB/p38 MAPK and the TLR7/NF-κB/IRF3 signaling pathways to induce proinflammatory cytokines and interferons, respectively, resulting in the orchestration of inflammatory and immune responses to the EV71 infection. Therefore, this study demonstrates that HRS acts as a key component of TLR7 signaling to orchestrate immune and inflammatory responses during EV71 infection, and provides new insights into the mechanisms underlying the regulation of host inflammation and innate immunity during EV71 infection.

## Author summary

Enterovirus 71 (EV71) is a highly infectious positive-stranded RNA virus that causes hand-foot-mouth disease (HFMD). As a major pathogen, EV71 infection leads to host immune responses in the disease severity. Toll-like receptors (TLRs) can recognize pathogens to induce host immunity and inflammation. Most TLRs must traffic from the endoplasmic reticulum (ER) to endolysosomal network before responding to ligands. The hepatocyte growth factor-regulated tyrosine kinase substrate (HRS) regulates ESCRT-0 complex and endosomal sorting of membrane proteins. HRS is required for ubiquitin-dependent TLR9 targeting to the endolysosome, however, the mechanism by which HRS regulates inflammation and immunity mediated by TLR7 is still largely unknown. Here, we reveal that HRS is a key component of TLR7 signaling to orchestrate immunity and inflammation during EV71 infection. EV71 infection induces the expression of HRS, which subsequently enhances the TLR7 complex formation by binding with TLR7 and TAB1. HRS facilitates TLR7/NF-κB/p38 MAPK and TLR7/NF-κB/IRF3 signaling pathways to produce proinflammatory cytokines and interferons, leading to induction of inflammatory and immune responses. Thus, we identify HRS as a key regulator of TLR7 signaling and illustrate a novel mechanism underlying the regulation of host immunity and inflammation during viral infection.

## Introduction

Upon infection, viral RNAs are recognized as pathogen-associated molecular patterns (PAMPs) by Toll-like receptors (TLRs) to trigger signaling events leading to the induction of interferons (IFNs) and proinflammatory cytokines [1, 2]. Most TLRs are intracellularly localized and must traffic from the endoplasmic reticulum (ER) to the endolysosomal network before they can respond to ligands [3–5]. Most RNA viruses (eg. hepatitis C virus, and vesicular stomatitis virus) activate TLR7, which is initiated by binding of TLR7 to the myeloid differentiation factor 88 (MyD88) adapter protein and interleukin-1 receptor-associated kinases (IRAK) and by recruiting tumor necrosis factor receptor-associated factor 6 (TRAF6), transforming growth factor 1-activated kinase-1 (TAK1) and TAK1-binding protein 1/2 (TAB1/2) [6, 7]. These events subsequently activate multiple signaling cascades, mitogen-activated protein kinase (MAPK), nuclear transcription factor-κB (NF-κB) and IFN regulatory factor 3/7 (IRF3/7), to induce the production of proinflammatory cytokines and IFNs, resulting in antiviral response and innate immunity [3, 8].

Enterovirus 71 (EV71) is a highly infectious positive-stranded RNA virus that causes hand-foot-mouth disease (HFMD), meningoencephalitis, neonatal sepsis, and even fatal encephalitis in children [9]. Although EV71 pathogenesis remains largely obscure, host immune responses play important roles in the disease severity [10]. EV71 infection induces the production of many proinflammatory cytokines that play important roles in disease development [11–13]. Serum concentrations of interleukin-1 (IL-1β), IL-1 receptor antagonist (IL-1Ra), and granulocyte colony-stimulating factor (G-CSF) are upregulated in EV71-infected patients with cardiorespiratory compromise [11]. The overdose of proinflammatory cytokines produced during EV71 infection is mediated by the activation of different TLRs [12]. EV71 infection induces TLR7 and TLR8 in epithelial cells to enhance the induction of IFN-beta [14]. EV71 infection also upregulates intestinal tract *TLR3*, *TLR4*, *TLR7*, and *TLR8* mRNA expression of children with severe HFMD [15]. However, the molecular mechanism by which EV71 infection induces TLRs-mediated inflammatory responses is still largely unknown.

Hepatocyte growth factor-regulated tyrosine kinase substrate (HRS) is a key component of the Endosomal Sorting Complexes Required for Transport (ESCRT-0) complex and required for endosomal sorting of membrane proteins into multivesicular bodies, lysosomes and vacuoles [16, 17]. HRS comprises an FYVE finger domain that facilitates HRS anchoring to the membrane and initiates its trafficking processes on endosomes [18]. Remarkably, HRS-mediated endosomal sorting benefits viral proteins trafficking in the host cell cytosol during viral envelopment and capsid secretion [19, 20]. During TLRs trafficking from the ER to endolysosomes, the intracellular co-factor UNC93B1 is indispensable for the control of this process [21, 22]. Similarly, HRS is required for ubiquitin-dependent TLR9 targeting to the endolysosome [4]. However, the detailed mechanism by which HRS regulates host inflammation and innate immunity during viral infection is still largely unknown.

In this study, we reveal that a set of cytokines is expressed differentially during EV71 infection. Clinical investigations, animal studies, and cell culture experiments demonstrate that EV71 activates TLR7 to induce inflammatory factors via p38 MAPK and NF-κB transcriptional factors. By genetic screening and clinical analysis, we identified HRS acting as an essential regulator of the TLR7 signaling. HRS facilitates the TLR7 complex assembly by binding to TLR7 and TAB1 during EV71 infection. In this process, HRS enhanced proinflammatory cytokines and interferons production dependent on the activation of TLR7/NF-κB/p38 and TLR7/NF-κB/IRF3 signaling pathways. Thus, we identify a new key regulator of TLR7 signaling and provide new insights into the regulation of host immunity during EV71 infection.

## Results

### EV71 induces proinflammatory cytokines in patients, mice, and cultured cells

**Fig 1:**
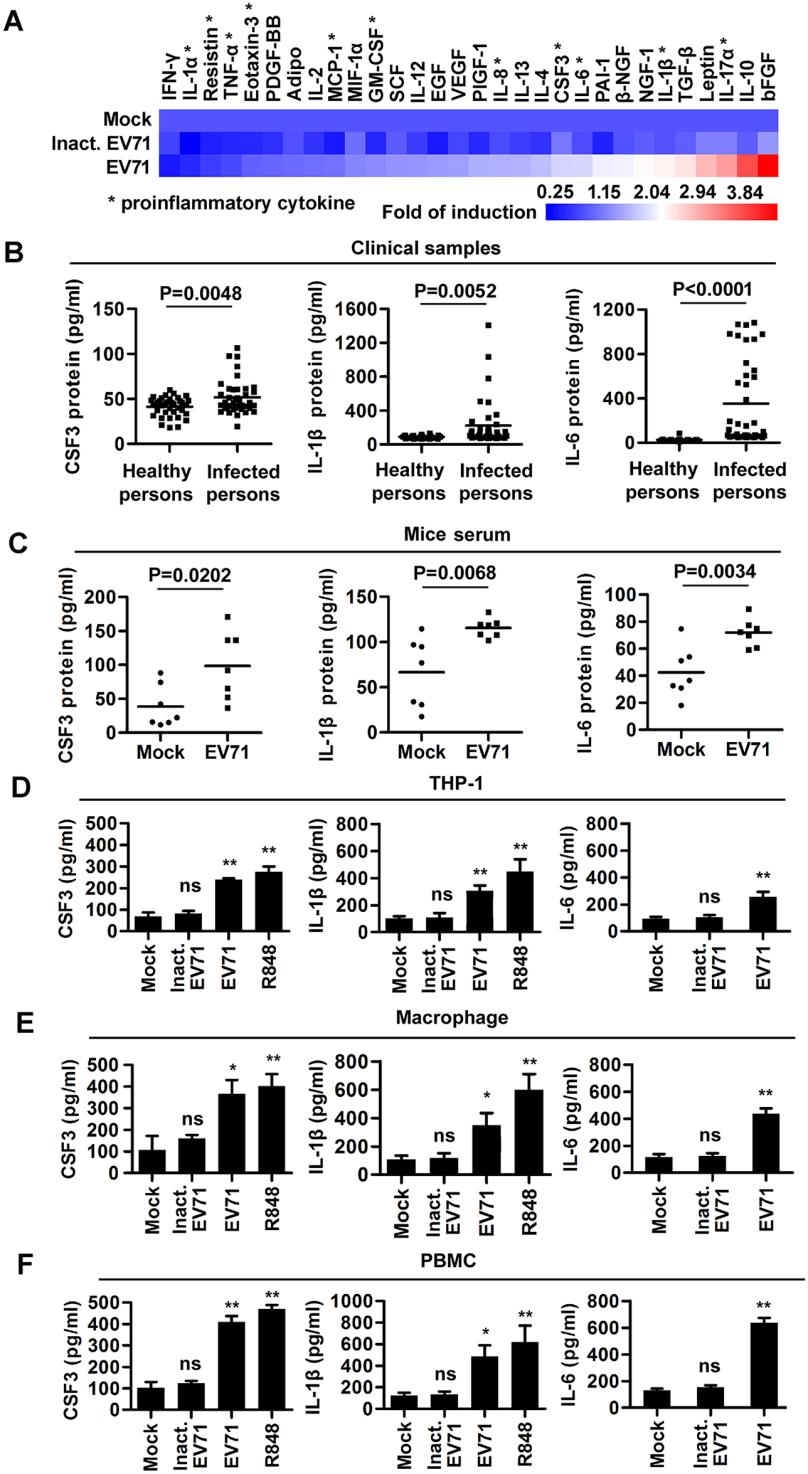
EV71 infection induces the productions of inflammatory cytokines *in vivo* and *in vitro*. (**A**) THP-1 derived macrophages were infected with EV71 (MOI = 5) or UV-inactivated EV71 for 12 h. The amount of 30 cytokines in cell supernatants was measured by Human Cytokine ELISA. Each cytokines concentrations in cell supernatants were reproducible and detectable. Data are shown as fold changes of protein expression compared to mock samples. Proinflammatory cytokines are marked by an asterisk. (**B** and **C**) CSF3, IL-1β, and IL-6 proteins in sera of EV71-infected patients (n = 40) and healthy individuals (n = 36) (**B**), or in sera of EV71-infected and mock-infected mice (each group, n = 7) (**C**) were measured by ELISA. (**D**–**F**) THP-1 cells (**D**), macrophages (differentiated from THP-1 cells) (**E**), and PBMCs (isolated from peripheral blood samples of healthy individuals) (**F**) were treated with 100 ng/ml R848 for 12 h or infected with UV-inactivated EV71 (Inact. EV71) or EV71 (MOI = 2) for 24 h. The supernatants of treated cells were collected and CSF3, IL-1β, and IL-6 levels were measured by ELISA. Data are shown as mean ± SD and correspond to a representative experiment out of three performed. ns, non-significant; *, *P* < 0.05; **, *P* < 0.01.

To investigate the mechanism underlying inflammation and immunity induced by EV71 infection, we initially determined the effect of EV71 on the production of proinflammatory cytokines. Human Cytokine ELISA Plate Arrays showed that EV71 infection induced production of 12 cytokines, including 3 proinflammatory factors as main biomarkers in clinical prognostics [11], colony stimulating factor 3 (CSF3), interleukin-1 beta (IL-1β), and IL-6; repressed production of 4 cytokines; and had no effect on 14 cytokines in human monocytic THP-1 cells (THP-1) (Fig 1A and S1 Table). The role of EV71 in activation of CSF3, IL-1β, and IL-6 was then confirmed. First, the production of CSF3, IL-1β, and IL-6 was analyzed in peripheral blood of 40 EV71-infected patients and 36 healthy individuals (Table 1 and S1A Fig). ELISA analyses indicated that CSF3, IL-1β, and IL-6 were present in higher amount in EV71 positive patients compared with healthy individuals (Fig 1B). Second, the expression of CSF3, IL-1β, and IL-6 was determined in EV71-infected mice. The viral VP1 RNA and protein were detected in peripheral blood mononuclear cells (PBMCs) and the spleen of infected mice (S1B and S1C Fig). Similar to patient serums, CSF3, IL-1β, and IL-6 protein were significantly induced in peripheral blood of EV71-infected mice (Fig 1C). Third, the production of CSF3, IL-1β, and IL-6 was evaluated in EV71-infected human acute monocytic leukemia cells (THP-1), differentiated macrophages derived from THP-1 cells (macrophages) [23] (S1D Fig), and human PBMCs cells. ELISA analyses revealed that CSF3, IL-1β, and IL-6 proteins were induced by EV71 or R848 (a TLR7 agonist) as a stimulus control [24], but not by mock-infection or UV-inactivated EV71, in THP-1, macrophages, and human PBMCs (Fig 1D–1F). Taken together, we demonstrate that EV71 induces CSF3, IL-1β and IL-6 both *in viv*o and *in vitro*.

**Table 1:** Demographic and baseline characteristics of EV71-negative individuals and EV71-positive patients^a^.

Characteristic	EV71-negative Individuals	EV71-positive Individuals
No. of case	36	40
No. of males/no. of females	22/14	23/17
Age (year)	2±1	1.9±1.2
Patients infected with EV71 genotype	NA	EV71 C4 subtype
EV71 RNA dose^b^	ND	0.083±0.010

^*a*^All EV71-positive individuals were confirmed to be negative for other enteroviruses and were not suffering from any concomitant illness, did not show any serological markers suggestive of autoimmune disease, and had not received any antiviral or immunomodulatory therapy prior to this study. Matched by sex and age, EV71-negative individuals with no history of HFMD were randomly selected as controls.

^*b*^EV71 RNA level in PBMCs is quantified using qPCR. The EV71 *VP1* RNA relative expression was normalized to *GAPDH* as an internal reference. NA, not available; ND, not detected.

### EV71 induces proinflammatory cytokines through TLR7 and NF-κB signaling

**Fig 2:**
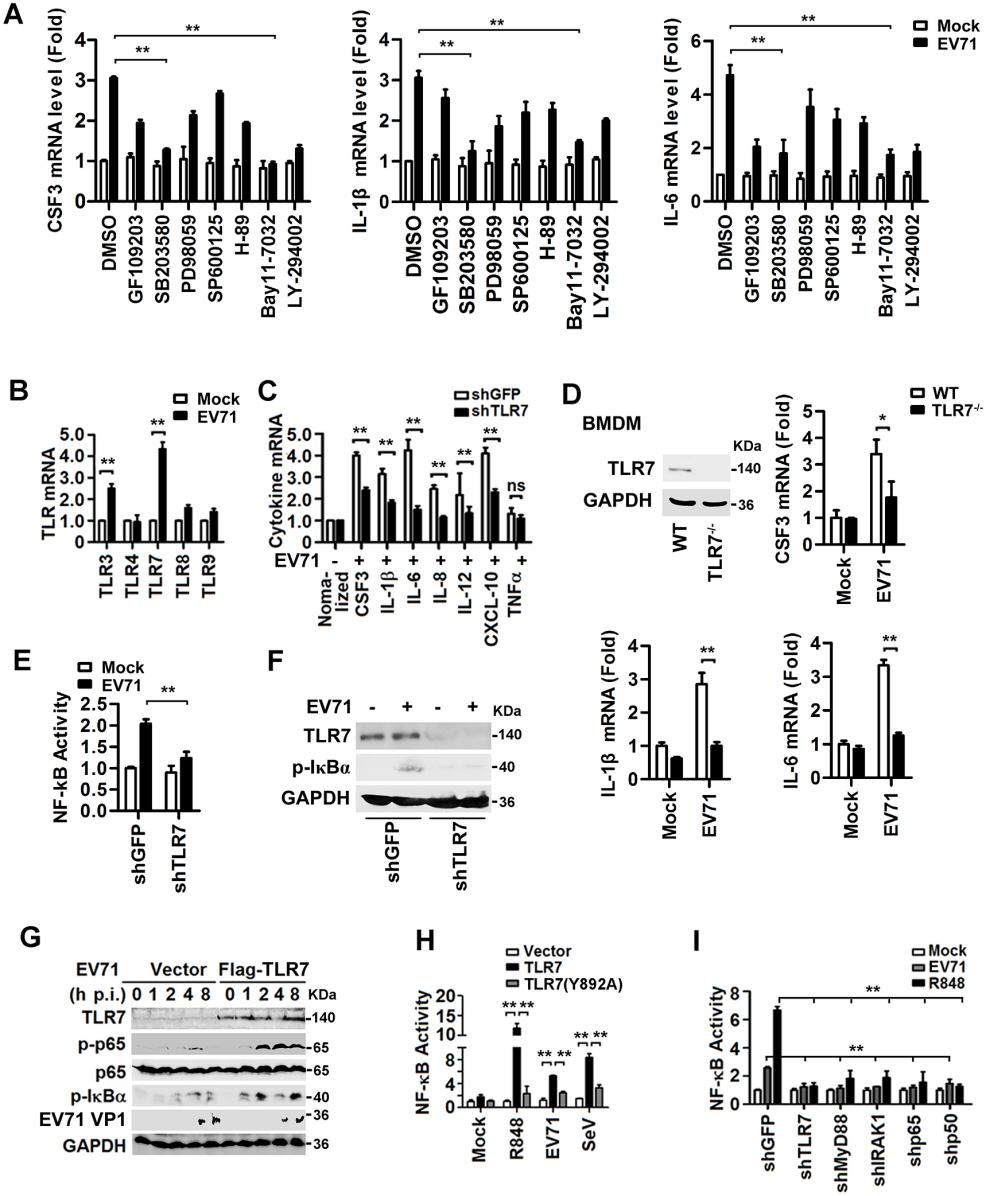
EV71 induces the production of proinflammatory cytokines *via* stimulating TLR7 signaling and NF-κB activity. (**A**) The monocytic THP-1 cells were treated with individual kinase inhibitors for 6 h, as indicated, and infected with EV71 (MOI = 5) for 12 h. The levels of *CSF3*, *IL-1β* and *IL-6* mRNAs were measured by qPCR. (**B**) THP-1 cells were infected with EV71 (MOI = 5) for 12 h and total mRNA extracts were prepared. The mRNA levels of TLRs expressed in the cells were determined by qPCR. (**C**) THP-1 cells were transfected with plasmid transcribing siRNA specific to *TLR7* (shTLR7) or its control (shGFP) and then infected with EV71 (MOI = 5) for 12 h. Cytokine mRNA levels were determined by qPCR. **(D**) Mouse bone marrow-derived macrophages (BMDMs) isolated from TLR7 wild-type (WT) or TLR7 knock-out (TLR7^-/-^) mice were infected with EV71 (MOI = 5) for 24 h. TLR7 protein was determined by Western blotting. Mouse *CSF3*, *IL-1β*, and *IL-6* mRNAs were measured by qPCR. (**E** and **F**) THP-1 cells were co-transfected with NF-κB reporter plasmid and siRNA specific to *TLR7* (shTLR7) or negative control (shGFP) and then infected with EV71 (MOI = 5) for 8 h. NF-κB activity was determined by luciferase activity assay (**E**). The endogenous protein levels were determined by western blotting with indicated antibodies (**F**). (**G**) HEK293T cells were transfected with pFlag2B (vector) or pFlag-TLR7 and infected with EV71 (MOI = 5). TLR7, p65, P-p65, EV71 VP1, and GAPDH proteins expressed in the treated cells were detected by Western blotting. (**H** and **I**) HEK293T cells were co-transfected with NF-κB reporter and pTLR7, pTLR7 (Y892A) or vector (**H**), or plasmids carrying shRNAs specific to indicated genes (**I**), and then stimulated with R848, or infected with EV71 (MOI = 5) or SeV (HA unit = 5) for 8 h, respectively. NF-κB activities were determined by luciferase activity assay. All qPCR assays used *GAPDH* mRNA as an internal control. Results were expressed as fold induction relative to control. ns, non-significant; *, *P* < 0.05; **, *P* < 0.01. Graphs show means ± SD, n = 3.

The mechanism by which EV71 activates proinflammatory cytokines was evaluated. Initially, signaling pathways required for EV71-mediated induction of CSF3, IL-1β, and IL-6 production were evaluated. THP-1 cells were infected with EV71 and treated with signaling component inhibitors that had no obvious effect on cell viability or EV71 replication except PD98059 that caused a reduction in viral protein (S2A and S2B Fig). *CSF3*, *IL-1β*, and *IL-6* mRNA levels were enhanced by EV71, however, in the presence of SB203580 (p38 MAPK inhibitor) or Bay11-7032 (NF-κB inhibitor), the induction of these mRNA levels were destroyed (Fig 2A). In the mouse macrophage-like Raw264.7 cell line, the same phenomenon was observed (S2C and S2D Fig). These results indicate that p38 MAPK and NF-κB are required for EV71-mediated induction of CSF3, IL-1β, and IL-6. Subsequently, the effect of EV71 on the expression of TLRs was determined. The expression of *TLR7* and *TLR3* mRNAs, but not *TLR4*, *TLR8* or *TLR9* mRNAs, was upregulated by EV71 in THP-1 cells (Fig 2B), suggesting that TLR3 and TLR7 may be involved in EV71-mediated induction of CSF3, IL-1β, and IL-6. Since it was previously reported that TLR3 signaling in macrophages is required for protection against EV71 infection in mice [25], we further evaluated the role of TLR7 in EV71-mediated activation of proinflammatory cytokines. We applied shRNA (short hairpin RNA) specific to *TLR7* (shTLR7) that could reduce TLR7 expression (S2E Fig). The mRNA levels of *CSF3*, *IL-1β*, *IL-6*, *IL-8*, *IL-12*, and *CXCL-10*, but not *TNFα*, were enhanced by EV71 infection, in case of shTLR7, mRNA levels of *CSF3*, *IL-1β*, *IL-6*, *IL-8*, *IL-12*, and *CXCL-10* were significantly reduced, suggesting that TLR7 is involved in these cytokines production (Fig 2C). To further explore the role of TLR7 in EV71-mediated induction of proinflammatory cytokines, mouse bone marrow-derived macrophages (BMDMs) were isolated from TLR7 WT mice and TLR7^-/-^ mice [26]. As expected, TLR7 protein was only detected in BMDMs of TLR7 WT mice, but not in BMDMs of TLR7^-/-^ mice (Fig 2D). *CSF3*, *IL-1β*, and *IL-6* mRNAs were induced by EV71 in BMDMs of TLR7 WT mice, but not induced by EV71 in BMDMs of TLR7^-/-^ mice (Fig 2D). Similarly, CSF3, IL-1β, and IL-6 proteins were also not induced by EV71 in BMDMs of TLR7^-/-^ mice (S2F Fig). These results confirmed that TLR7 is required for EV71-mediated induction of proinflammatory cytokines.

Since TLR7 and NF-κB are required for the induction of CSF3, IL-1β, and IL-6 production, we evaluated the correlation between TLR7 and NF-κB. First, NF-κB activity was induced by EV71 infection, but this induction was reduced in the presence of shTLR7 (Fig 2E), as well as EV71-induced phosphorylation of endogenous IκBα (Fig 2F). These results suggest that TLR7 is required for EV71-induced activation of NF-κB. Correspondingly, phosphorylation of NF-κB p65 and IκBα was enhanced in cells containing TLR7 upon EV71-infection, but not in mock-infected cells (Fig 2G), indicating that TLR7 activates NF-κB during viral infection. Third, plasmids expressing TLR7 and the inactive TLR7 mutant Y892A were constructed and the expression of TLR7 and TLR7 (Y892A) proteins was confirmed (S2G Fig). NF-κB activity was stimulated by TLR7, but not by TLR7 (Y892A), in the cells treated with R848 or infected with EV71 or SeV (Fig 2H), implicating that TLR7 plays an essential role in EV71-mediated activation of NF-κB. Finally, a HEK293T cell line was generated that steadily expressed TLR7 and NF-κB. In this stable cell line, NF-κB activity was enhanced by EV71 infection and R848 stimulation, but attenuated in the presence of shTLR7, shMyD88, shIRAK1, shNF-κB p65, and shNF-κB p50 (Fig 2I), confirming that TLR7, MyD88, and IRAK1 are involved in the activation of NF-κB during viral infection. Thus, these results reveal that EV71 activates TLR7 signaling to stimulate NF-κB, leading to induction of proinflammatory cytokines.

### Expression of HRS is induced and correlated with TLR7 expression to activate TLR7/NF-κB signaling upon EV71 infection

**Fig 3:**
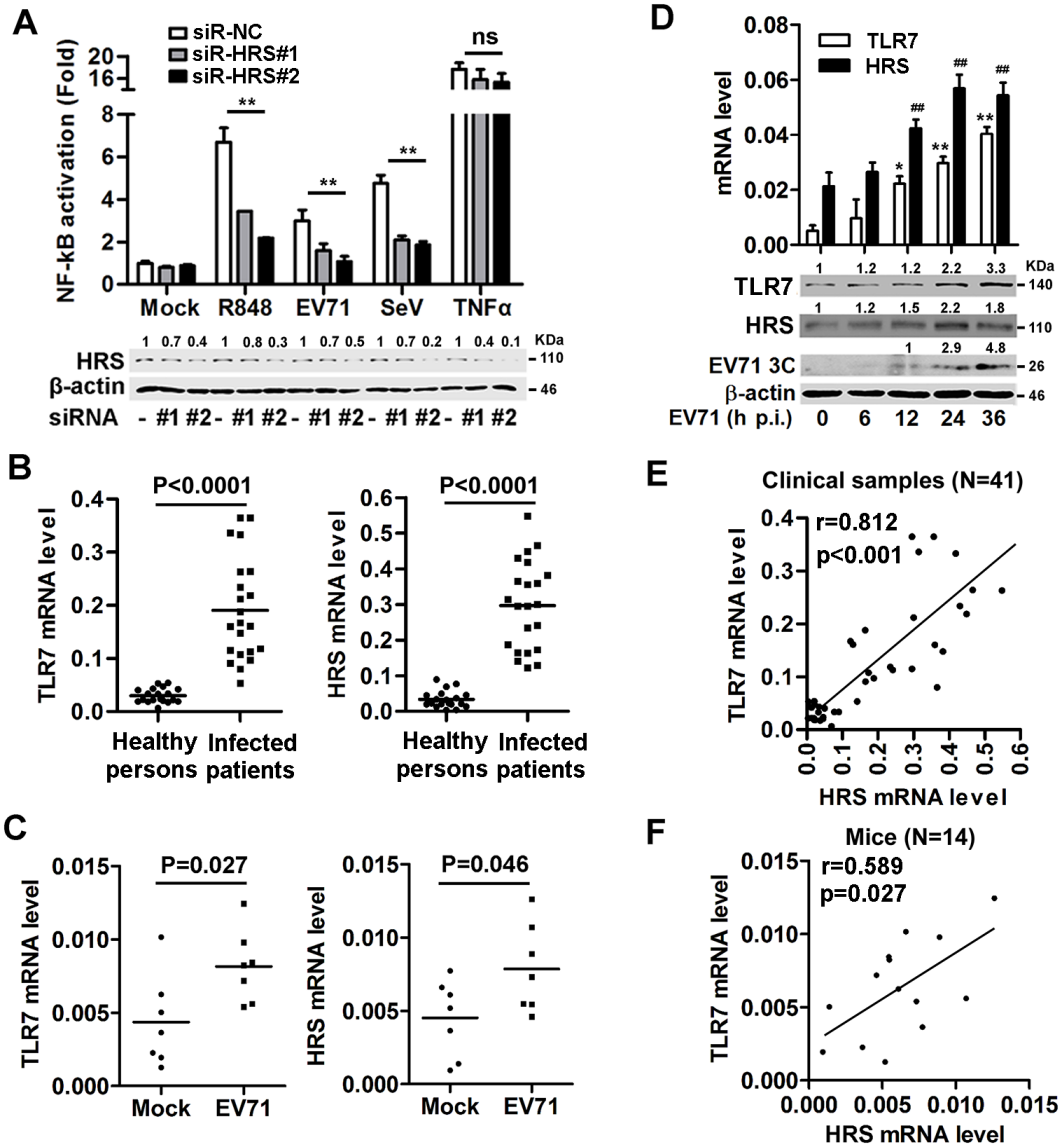
TLR7 expression correlates with HRS expression during EV71 infection. (**A**) Stable HEK293T/TLR7/NF-κB reporter cells were transfected with siR-NC, siR-HRS#1 and siR-HRS#2, treated with R848 or TNFα, and infected with EV71 or SeV. NF-κB activities were determined by luciferase activity assays. The HRS and β-actin proteins expressed in the treated cells were detected by Western blotting. The indicated band intensity represents as fold changes to internal control by using Image J software analysis. Results were expressed as fold induction relative to control. ns, non-significant; *, P < 0.05; **, P < 0.01. Graphs show means ± SD, n = 3. (**B** and **C**) PBMCs were isolated from EV71-infected patients (n = 22) and healthy individuals (n = 19) (**B**) or EV71-infected (n = 7) and mock (infected with PBS) mice (n = 7) (**C**), and then total RNA extraction was performed. The *TLR7* and *HRS* mRNA levels were determined by qPCR with *GAPDH* mRNA as an internal control. (**D**) THP-1 cells were infected with EV71(MOI = 5) for different times, as indicated. *TLR7* and *HRS* mRNA (upper panel), TLR7 and HRS protein (lower panel) were measured by qPCR and Western blotting analyses, respectively. **, *P* < 0.01 for TLR7; ^##^, *P* < 0.01 for HRS. (**E** and **F**) Pearson’s correlation of *HRS* and *TLR7* mRNA levels was analyzed in clinical samples (**E**) or mice (**F**).

To determine the mechanism by which EV71 activates TLR7/NF-κB, novel cellular factors involved in such activation were identified. A bioinformatics approach was employed to integrate a protein-protein interaction network in STRING databases [27], which comprised known and predicted proteins associated with the TLR7 signaling pathway. By using this approach, we predicted that 14 out of 19 cellular factors were potential TLR7-associated proteins (S3A Fig). We further conducted an RNAi mini-library screening to assess the functional integration of these factors in TLR7 signaling (S2 Table). HEK293T/TLR7/NF-κB cells were transfected with an array of siRNAs targeting to these genes. NF-κB activity was stimulated by R848, and significantly repressed by siRNAs to HRS (siR-HRS) (S3B Fig), suggesting that HRS is involved in regulation of TLR7/NF-κB. Requirement of HRS for TLR7 signaling was further evaluated by using siRNA, which attenuated HRS expression (S3C Fig), but had no effect on cell viability or cell apoptosis (S3D and S3E Fig). NF-κB activity was upregulated in HEK293T/TLR7/NF-κB cells stimulated with R848 or TNFα or infected with EV71 or SeV, whereas the activation mediated by R848 stimulation, EV71 or SeV infection was reduced in the presence of siR-HRS, but TNFα-mediated activation was not affected in the presence of siR-HRS (Fig 3A).

Since EV71 activates TLR7 signaling and HRS participates in this signaling, the effect of EV71 on HRS production was investigated. *TLR7* and *HRS* mRNAs were significantly induced in EV71-infected patients and mice PBMCs (Fig 3B and 3C) and TLR7 and HRS mRNAs and proteins were stimulated in EV71-infected THP-1 cells (Fig 3D), demonstrating that HRS production is induced by EV71 infection in patients, mice, and cultured cells. Since EV71 induces TLR7 and HRS, the correlation between TLR7 and HRS during viral infection was determined. *TLR7* and *HRS* mRNA levels were correlated well in EV71-infected patients and mice PBMCs (Fig 3E and 3F), revealing that TLR7 production is positively correlated with HRS expression during EV71 infection. The effect of EV71 infection on HRS expression was explored. By using bioinformatic approaches, we predicted several NF-κB subunit binding sites in both human and mouse *HRS* and *TLR7* promoters (S4A Fig). In Raw264.7 cells, EV71 enhanced both HRS and TLR7 protein expression, but this induction was abrogated in the presence of NF-κB inhibitor Bay11-7032 (S4B Fig). Considering that EV71 infection facilitates TLR7-mediated NF-κB activation, we determined whether TLR7 plays a role in EV71-induced HRS expression. EV71 facilitated HRS expression in BMDMs of TLR7 WT mice, but failed to enhance HRS expression in BMDMs of TLR7^-/-^ mice (S4C Fig), indicating that EV71 induces HRS production through TLR7 signaling. Taken together, our results suggest that HRS expression is induced by EV71 and the levels of TLR7 and HRS are correlated *in vitro* and *in vivo* upon EV71 infection.

### HRS interacts and co-localizes with TLR7 and TAB1

**Fig 4:**
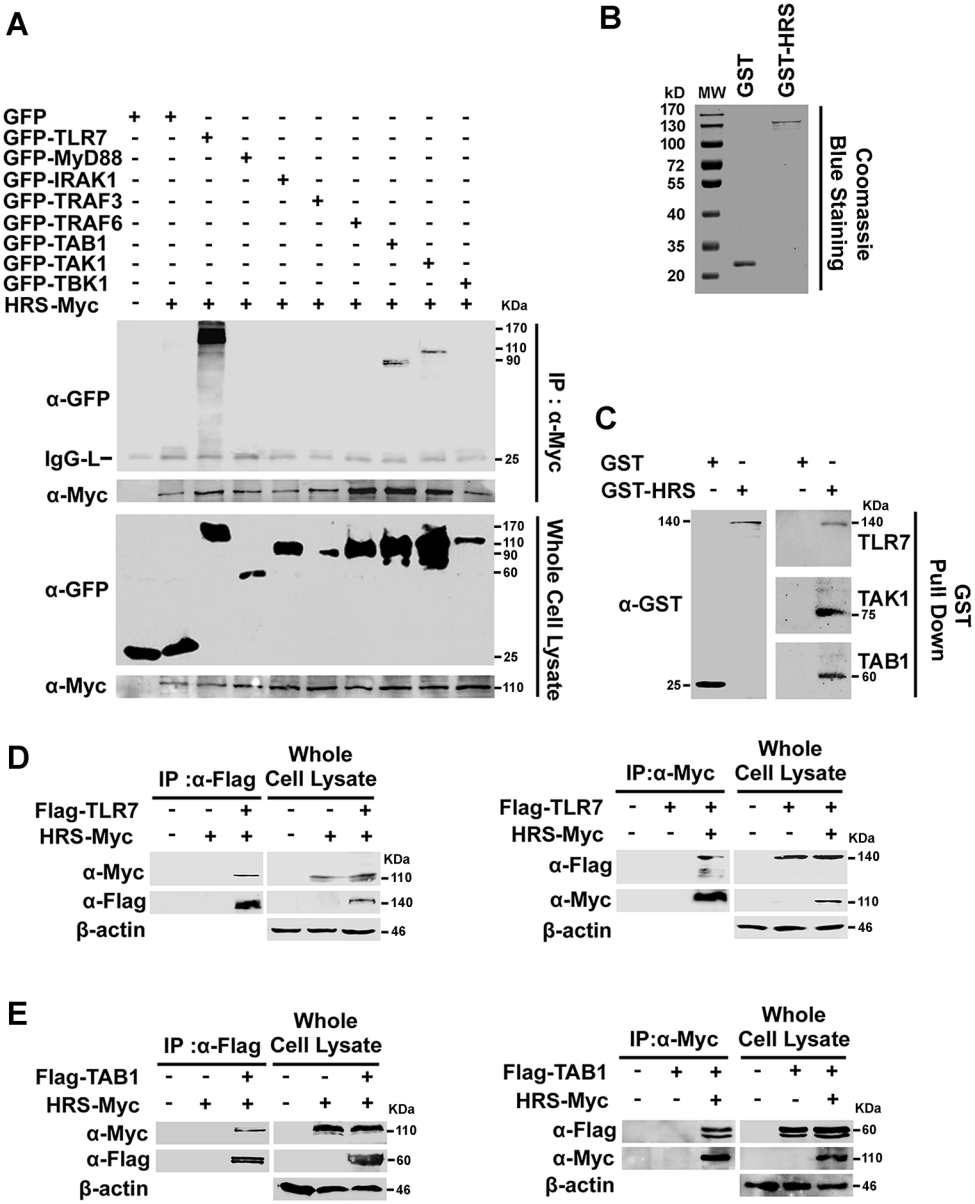
HRS interacts with TLR7 and TAB1. (**A**) HEK293T cells were co-transfected with plasmids expressing GFP protein or GFP fusion proteins as indicated and plasmid expressing HRS-Myc fusion protein. Whole cell lysates were prepared and immunoprecipitated using anti-Myc antibody (α-Myc). Immunoprecipitates were assayed using Western blotting analysis with specific antibodies. GFP (α-GFP), Myc (α-Myc) and IgG light chain (IgG-L). (**B** and **C**) GST and GST-HRS proteins were expressed, purified, subjected to SDS-PAGE, and detected with Coomassie blue staining (**B**), and subsequently were incubated with THP-1 cell lysates. Complexes were pulled down using GST beads and detected using immunoblotting (**C**) Immunoprecipitates were assayed by Western blotting with a GST specific antibody. (**D** and **E**) HEK293T cells were co-transfected with plasmid expressing Flag-TLR7 and HRS-Myc (**D**), or Flag-TAB1 and HRS-Myc (**E**). Cell lysates were prepared and immunoprecipitated using α-Flag or α-Myc antibodies.

**Fig 5:**
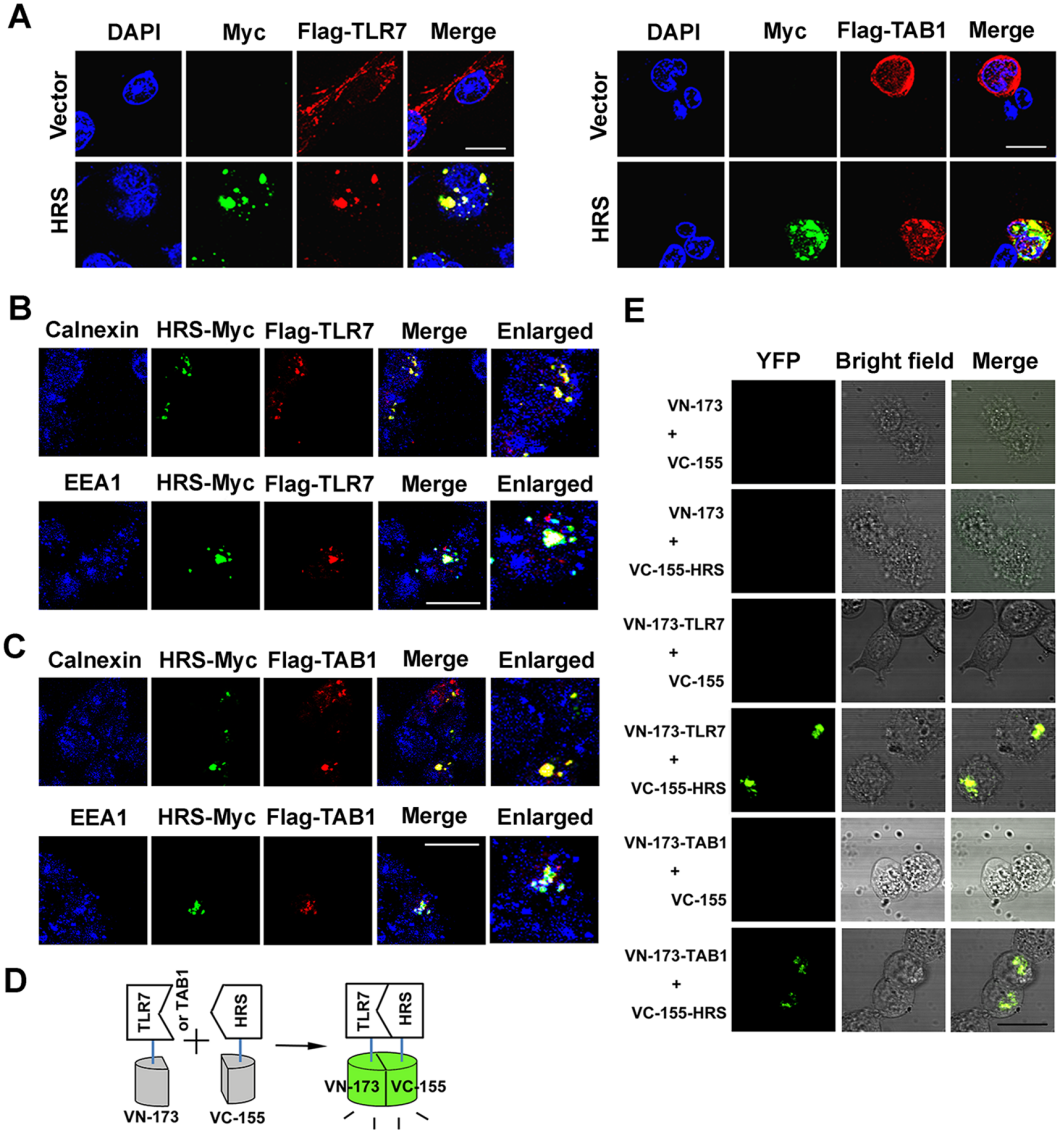
HRS co-localizes with TLR7 and TAB1. (**A**) HEK293T cells were co-transfected with plasmids expressing HRS-Myc and Flag-TLR7 (upper panel) or Flag-TAB1 (lower panel) and probed with indicated antibodies before confocal microscopy. Bar = 20 μm. (**B** and **C**) HEK293T cells were co-transfected with plasmid expressing Flag-TLR7 and HRS-Myc (**B**) or Flag-TAB1 and HRS-Myc (**C**). Cells were probed with mouse anti-Myc (Green), rat anti-Flag (Red), and rabbit anti-EEA1 or Calnexin (Blue) antibodies before confocal microscopy. Bar = 20 μm. (**D** and **E**) Bimolecular fluorescence complementation (BiFC) assays for detection of physical interactions (**D**) between HRS and TLR7 or TAB1 (**E**). HEK293T cells were co-transfected with empty vector or plasmids expressing VC-155-HRS and VN-173-TLR7 or VN-173-TAB1 fusion protein. At 24 h post-transfection, living cells were observed by confocal microscopy. Bar = 20 μm.

HRS acts as a mediator in endosome trafficking by binding to targeted proteins [28] and is required for TLR7-and TLR9-dependent innate immune responses [4]. To identify novel HRS-interacting factors involved in TLR7 signaling, HEK293T cells were co-transfected with plasmids expressing HRS-Myc and individual GFP-tagged proteins. Co-immunoprecipitation (Co-IP) showed that HRS interacted with TLR7, TAB1, and TAK1, but not MyD88, IRAK1, TRAF3, TRAF6, or TBK1 (Fig 4A). Interactions of HRS with TLR7, TAB1, and TAK1 were verified by GST pull-down assays with purified GST and GST-HRS proteins (Fig 4B and 4C). To confirm such interactions of HRS with TLR7 and TAB1, HEK293T cells were co-transfected with pHRS-Myc and pFlag-TLR7 or pFlag-TAB1. Co-IP results further confirmed that HRS interacted with TLR7 and TAB1 (Fig 4D and 4E). The associations of HRS with TLR7 and TAB1 were also examined by confocal microscopy. TLR7 and TAB1 were distributed diffusely in the cytoplasm in the absence of HRS, but co-localized with HRS and redistributed to loci in the cytoplasm in the presence of HRS (Fig 5A). We also showed that the interaction of HRS with TLR7 and with TAB1 was occurred in the endosomes (as indicated by EEA1, an endosome marker), but not in the endoplasmic reticulum (as indicated by Calnexin, an ER marker) [29] (Fig 5B and 5C).

The interactions of HRS with TLR7 and TAB1 were further confirmed by bimolecular fluorescence complementation (BiFC) analysis in living cells (Fig 5D). Fluorescence was not detected in control experiments when only one fusion protein was expressed with the complementary YFP fragment alone. Strong fluorescence was observed between VC-155-HRS with VN-173-TLR7 or VC-155-HRS with VN-173-TAB1 fusion proteins (Fig 5E), suggesting that direct interactions occurred between HRS and TLR7 or between HRS and TAB1. These results demonstrate that HRS interacts and co-localizes with TLR7 and TAB1.

### HRS binds to TLR7 and TAB1 to participate in the TLR7 complex during viral infection

**Fig 6:**
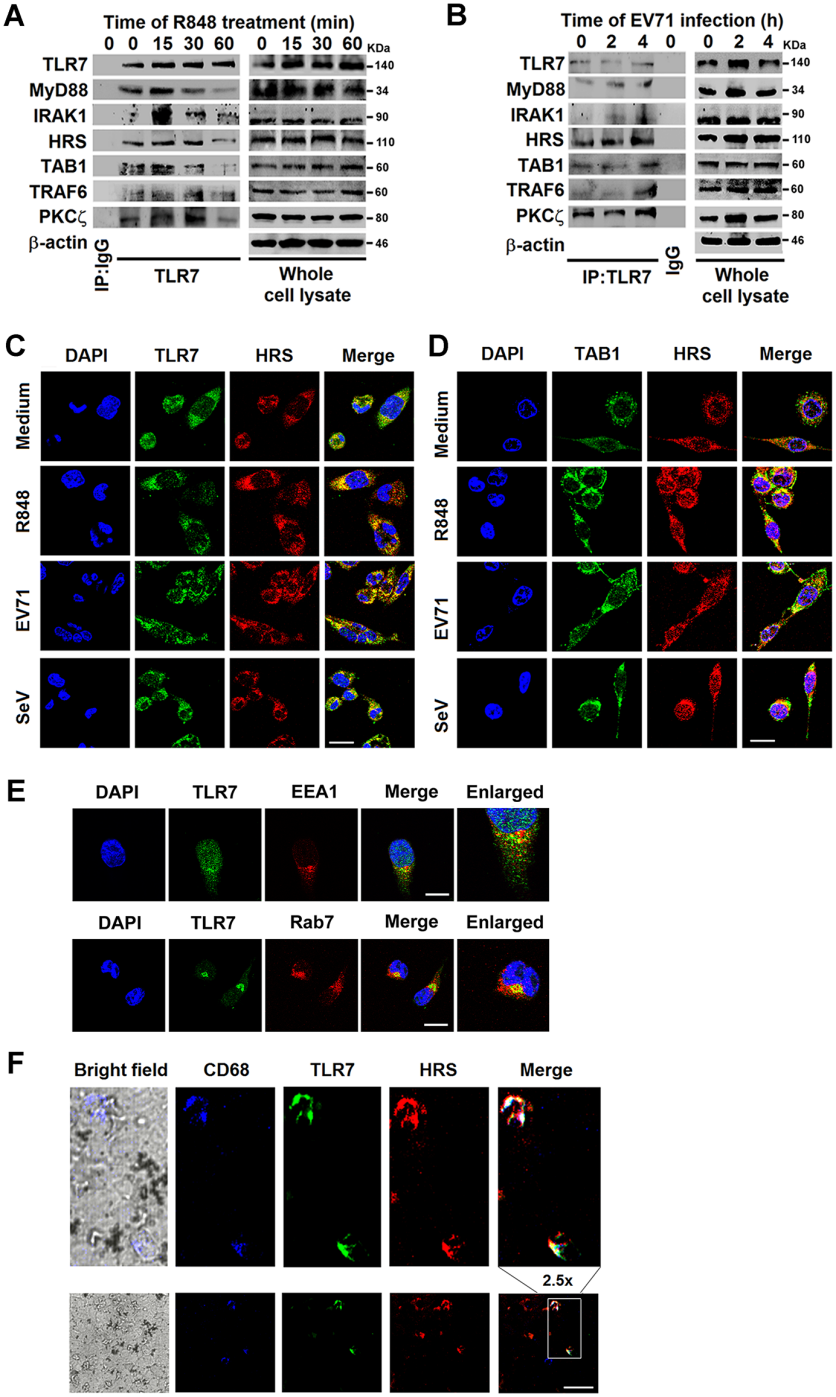
HRS interacts with TLR7 and TAB1 in the TLR7 complex during viral infection. (**A** and **B**) Macrophages derived from THP-1 cells were stimulated with R848 (100 ng/ml) (**A**) or EV71 (MOI = 5) (**B**) for various time periods as indicated. Cell lysates were prepared and immunoprecipitated with rabbit anti-TLR7 or rabbit anti-IgG antibodies. Immunoprecipitates were assayed using Western blotting analysis with specific antibodies as indicated. (**C** and **D**) Macrophages were stimulated with R848 for 15 min, or infected with EV71 or SeV for 4 h, and then probed with indicated antibodies before confocal microscopy. Bar = 20 μm. (**E**) Macrophages were treated with R848 (100 ng/ml) for 30 min. Cells were probed with indicated antibodies and DAPI stain before confocal microscopy. Bar = 20 μm. (**F**) Mice were infected with EV71 and sacrificed at indicated period. Mice spleens were subjected to immunofluorescence (IF) staining with HRS (Red), TLR7 (Green), and CD68 (Blue) antibodies. Bar = 20 μm.

To determine the function of HRS in TLR7 signaling, we evaluated the associations between HRS and the components of the TLR7 complex in differentiated macrophages derived from THP-1 cells. We found that HRS, together with MyD88, IRAK1, TAB1, TRAF6, and PKCζ proteins, could be coimmunoprecipitated with TLR7 in R848-stimulated (Fig 6A) or EV71-infected (Fig 6B) macrophages, suggesting that HRS may be a component of the TLR7 complex. To confirm the involvement of HRS in TLR7 signaling, distributions of HRS and TLR7 in macrophages were visualized by confocal microscopy. Endogenous TLR7 and HRS (Fig 6C) as well as endogenous TAB1 and HRS (Fig 6D) were distributed diffusely in the cytoplasm, but large proportions of TLR7 and HRS co-localized at foci in specific areas in the cytoplasm of R848-stimulated, EV71-infected or SeV-infected macrophages. Additionally, TLR7 mainly co-localized with EEA1 (an early endosome marker) and Rab7 (a late endosome marker) [29] in R848-stimulated macrophages (Fig 6E). These results reveal that HRS co-localized with TLR7 and TAB1 during viral infection, and it most likely locates to the endosomes.

Moreover, we evaluated the colocalization of HRS and TLR7 in the spleens of EV71-infected TLR7 WT mice or TLR7^-/-^ mice. As expected, TLR7 protein was only detected in the spleens of TLR7 WT mice (S5A Fig, left), but not in the spleens of TLR7^-/-^ mice (S5A Fig, right). CD68 positive cells were not produced in the spleen of mock-infected TLR7 WT mice (S5B Fig, left), but produced in the spleens of EV71-infected TLR7 WT mice (S5B Fig, right), suggesting that inflammatory response is induced by EV71 infection *in vivo* [30]. Moreover, HRS co-localized with TLR7 in CD68 positive cells in the spleens of EV71-infected TLR7 WT mice (Fig 6F). Taken together, we show that HRS binds and co-localizes with TLR7 *in vitro* and *in vivo*, and may act as a component in the TLR7 complex.

### HRS recruits TLR7 in early- and late-endosome to facilitate the TLR7 complex assembly

**Fig 7:**
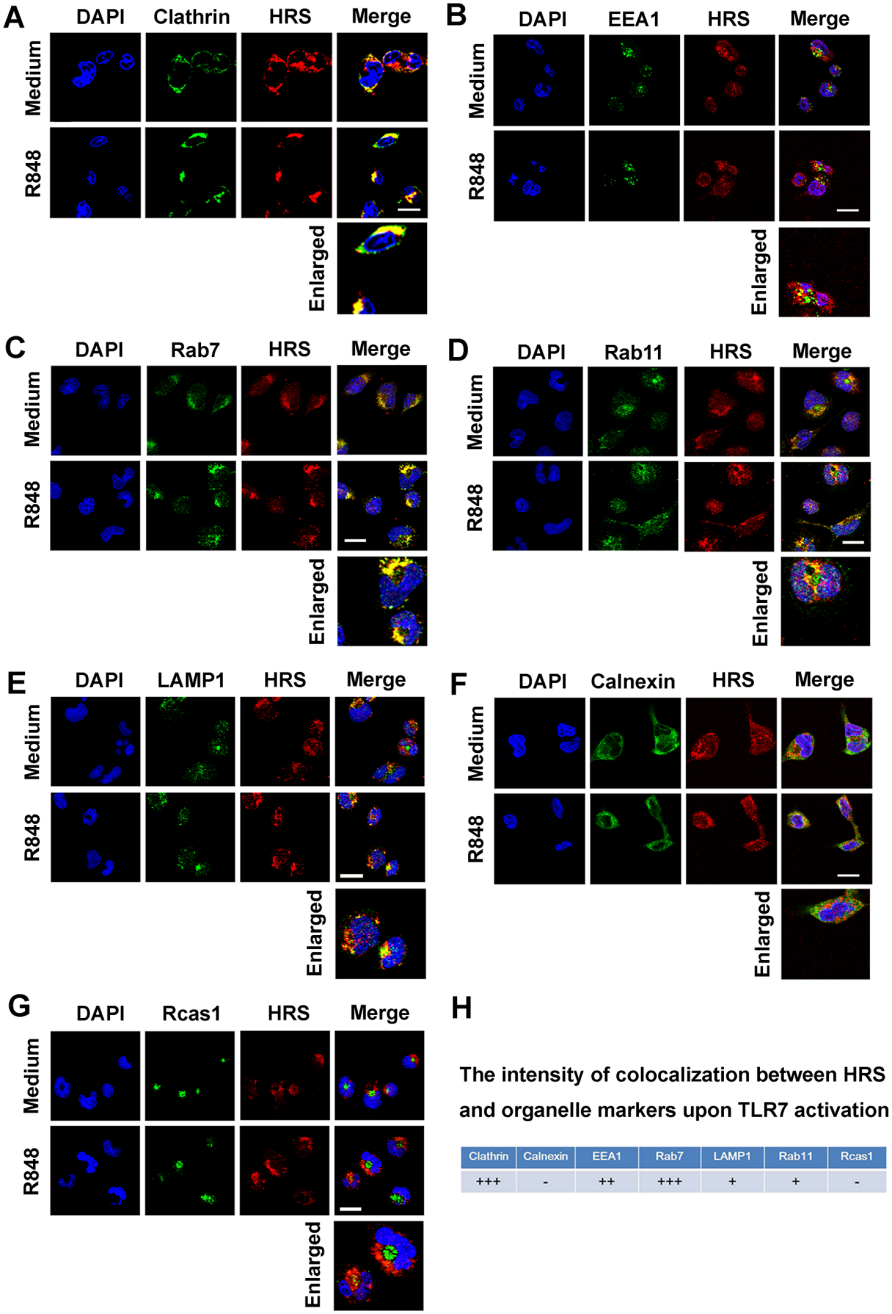
The sub-cellular localization of HRS during TLR7 signaling activation. **(A–G)** Macrophages were stimulated without or with R848 for 15 min, and then probed with HRS or organelles markers antibodies against clathrin (**A**), EEA1 (**B**), Rab7 (**C**), Rab11 (**D**), LAMP1 (**E**), Calnexin (**F**) or Rcas1 (**G**) before confocal microscopy, Bar = 20 μm. **(H)** The intensity of colocalization was calculated by using Image J software analysis. +++, >80% colocalization; ++, 60%~80% colocalization; +, 40%~60% colocalization; -, < 40% colocalization.

The role of HRS in the regulation of the TLR7 complex was investigated by examining whether HRS is involved in dynamic endosomal events upon TLR7 activation. Endogenous clathrin and HRS did not co-localize in the cytoplasm of untreated macrophages, but most clathrin and HRS co-localized at foci in particular areas in the cytoplasm of R848-treated cells (Fig 7A). Since clathrin participates in endosomal trafficking [31], this result suggests that HRS may regulate TLR7 endosomal trafficking. TLRs must move from the ER to the Golgi to endosomes during intracellular stimulation [32, 33]. We showed that TLR7 mainly co-localized with EEA1 and Rab7 (endosome markers) in R848-stimulated cells (Fig 6E). Accordingly, when TLR7 signaling was activated by R848, HRS mainly co-localized with EEA1 (Fig 7B) and Rab7 (Fig 7C), but not with Rab11 (a marker of recycling endosome) (Fig 7D), LAMP1 (a marker of lysosome) (Fig 7E), Calnexin (Fig 7F) or Rcas1 (Fig 7G) (a Golgi marker) [29, 34] in macrophages (Fig 7H), these observations suggest that HRS locates to endosomes upon TLR7 activation.

**Fig 8:**
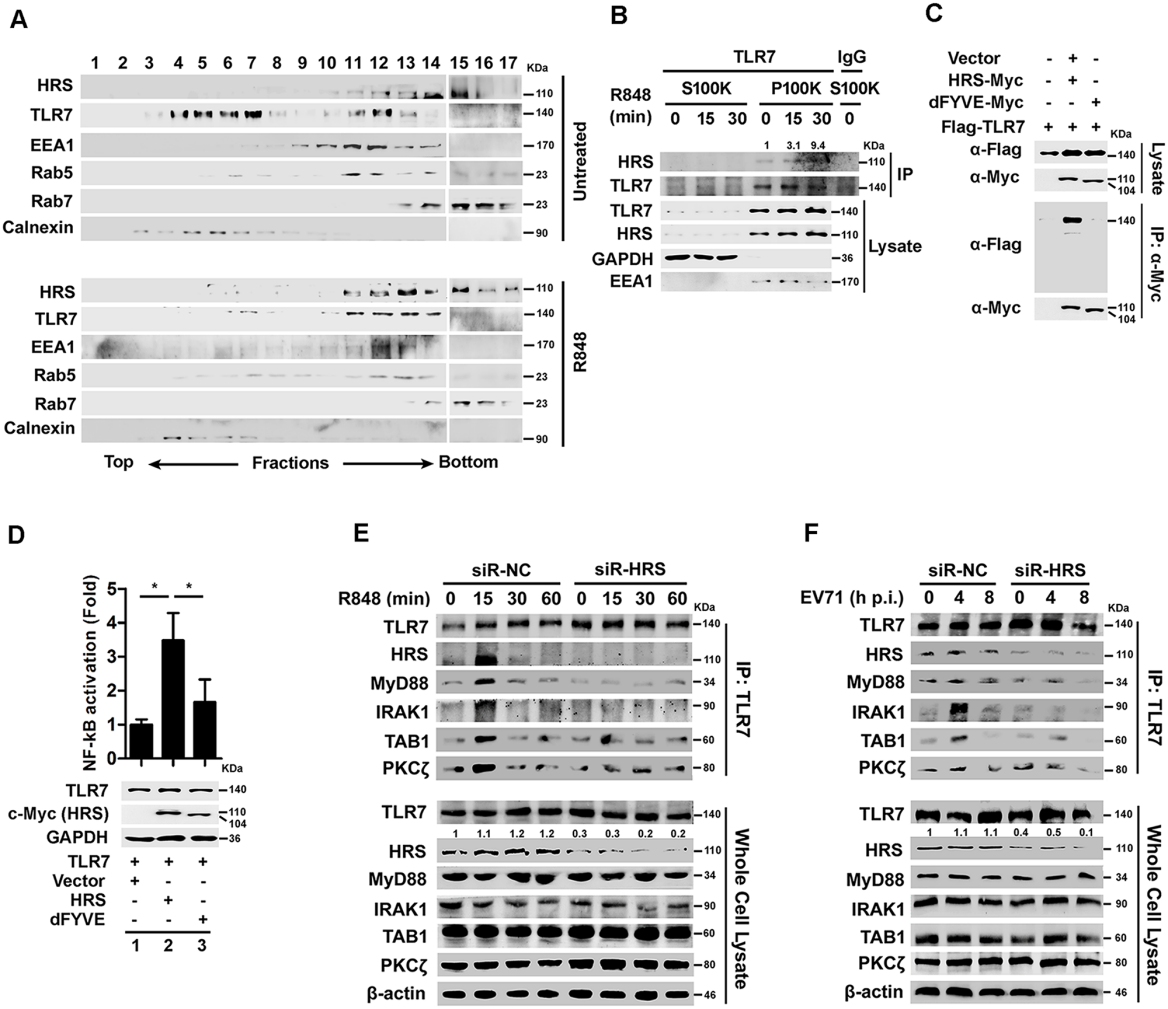
HRS interacts with TLR7 to facilitate signaling activation. (**A**) Macrophages were harvested after treated without or with R848 (100 ng/ml) for 30 min. Samples were homogenized and resolved by ultracentrifugation. Fractions were collected from the top of the gradient and analyzed for the distribution of indicated intracellular proteins by Western blotting. (**B**) Macrophages were treated with R848 (100 ng/ml) for indicated time and then applied for cell fraction. Separated cell fractions were subjected to immunoprecipitation (IP) and analyzed by Western blotting. S100K and P100K indicate supernatant and precipitate of cell fractions after 100,000 *g* ultracentrifugation, respectively. Indicated band intensity represents as fold changes to immunoprecipitated TLR7 by using Image J software analysis. (**C**) HEK293T cells were co-transfected with plasmid expressing Flag-TLR7 and HRS-Myc or FVYE domain deleted HRS-Myc (dFYVE-Myc). Whole cell lysates were prepared and immunoprecipitated and then analyzed by Western blotting. (**D**) HEK293T/TLR7/NF-κB reporter cells were transfected with plasmids expressing HRS or HRS deletion. NF-κB activities were determined using luciferase activity assays. The proteins expressed in the treated cells were detected by Western blotting. *, *P* < 0.05. Graphs show means ± SD, n = 3. (**E** and **F**) Macrophages derived from THP-1 cells were stimulated with R848 (**E**) or EV71 (**F**) for various time periods as indicated. Cell lysates were prepared and immunoprecipitated with rabbit anti-TLR7 or rabbit anti-IgG antibodies and then analyzed by Western blotting.

The subcellular compartments distinct from endocytic vesicles were then separated. In the absence of the TLR7 ligand R848, TLR7 was present in fractions 3 to 7 as the ER manifested by Calnexin, whereas HRS was present in fractions 10 to 16. In the presence of R848, both TLR7 and HRS were present in fractions 10 to 16, the early- and late- endosome marker proteins EEA1 and Rab7 were also present in fractions 10 to 16 (Fig 8A), these findings suggest that HRS and TLR7 locate to the same components only upon TLR7 stimulation. The separated fractions were further analyzed by Co-IP as described previously [35]. TLR7 and HRS were detected only in the membrane fraction (P100K), but not cytosol fraction (S100K) (Fig 8B, lysate), and the interaction of TLR7 with HRS was enhanced by R848 stimulation (Fig 8B, IP), suggesting that TLR7 and HRS located in the membrane upon TLR7 activation. In addition, HRS interacted with TLR7, but HRS-dFYVE (a mutant HRS with the FYVE domain deleted) failed to interact (Fig 8C), revealing that this domain is required for the interaction between TLR7 and HRS. Since FYVE domain is essential for the binding of HRS to the endosomal membrane [18], our results suggest that HRS binds to TLR7 at the endosomal membrane. Moreover, NF-κB activity was stimulated by coexpression of TLR7 and HRS, but not by TLR7 with HRS-dFYVE (Fig 8D), indicating that the FYVE domain of HRS is required for TLR7-mediated activation of NF-κB. Thus, HRS may recruit TLR7 in early- to late-endosomes to facilitate TLR7 activation, and the interaction between HRS and TLR7 may be important for TLR7 complex formation in endosomes.

Induction of TLR7 signaling is initiated by MyD88 and IRAK binding that further recruits TRAF6, TAK1, and TAB1 to activate multiple intracellular signaling cascades, including MAPK, NF-κB, and IRF3/7, leading to the production of proinflammatory cytokines [3, 36]. Since proinflammatory cytokines are predominantly produced by macrophages and dendritic cells (DCs) [37], we determined the role of HRS in the regulation of the TLR7 complex in macrophages. In R848-stimulated macrophages (Fig 8E) or EV71-infected macrophages (Fig 8F), the interactions of TLR7 with MyD88 and IRAK1 were greatly reduced in cells transfected with siR-HRS. However, unexpectedly, the interaction between TLR7 and TAB1 and PKCζwas not reduced in HRS knock-down cells (Fig 8E and 8F). These results suggest that HRS is important for the interaction of the TLR7 with MyD88 and IRAK1.

### HRS is important for TLR7-mediated cytokine induction *in vitro* and *in vivo*

**Fig 9:**
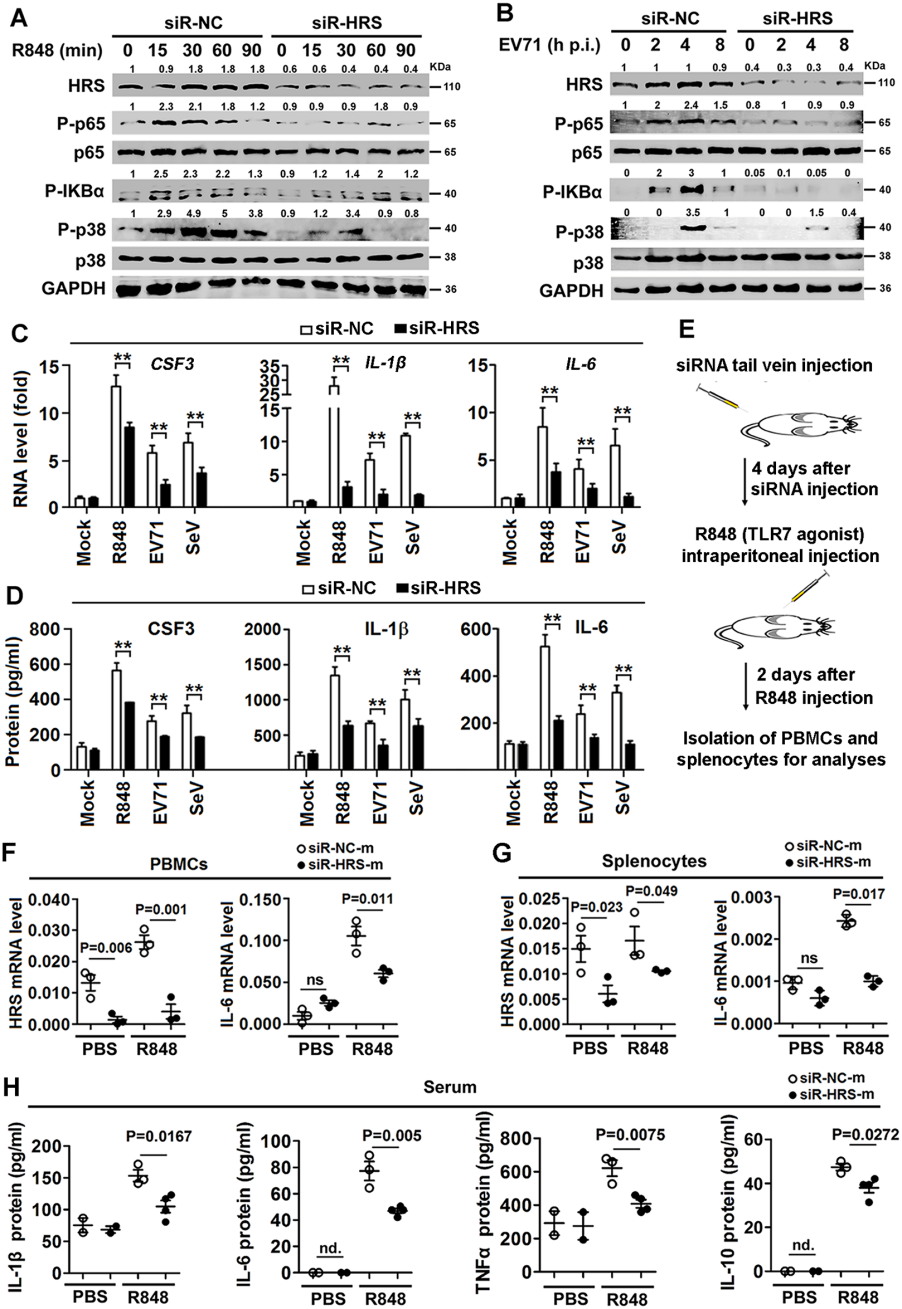
HRS is important for TLR7-mediated proinflammatory cytokines production during viral infection. (**A** and **B**) Macrophages were transfected with siR-HRS or siR-NC and then stimulated with R848 (A) or infected with EV71 (**B**) for various time periods as indicated. Cell extracts were prepared and the proteins in the cell lysates were detected using Western blotting analyses with corresponding antibodies. The indicated band intensity represents as fold changes to internal control GAPDH. (**C** and **D**) Macrophages were transfected with siR-NC or siR-HRS and stimulated with R848 or infected with EV71 or SeV. The levels of CSF3, IL-1β, and IL-6 mRNAs and proteins were determined using qPCR (**C**) and ELISA (**D**), respectively. **, *P*<0.01. Graphs show means ± SD, n = 3. (**E**–**H)** Mice were treated with 5’-cholesterol-modified siRNA duplex targeted to HRS (siR-HRS-m) and its control (siR-NC-m) *via* tail vein injection and injected intraperitoneally with R848; PBMCs and splenocytes were isolated from the mice. IL-6 mRNA levels expressed in mouse PBMCs (**F**) and splenocytes (**G**) were determined using qPCR (each group, n = 3). The proinflammatory cytokines proteins in mouse serum (**H**) were determined using ELISA (n = 2–4). ns, not significant; nd., not detected. Data show means ± SEM (standard error of the mean).

Because HRS is important for TLR7 activation, the effect of HRS on downstream events of TLR7 signaling was examined. p-p65, p-IκBα, and p-p38 were reduced in siR-HRS transfected cells upon R848 stimulation (Fig 9A) and EV71 infection (Fig 9B), revealing that HRS plays a critical role for TLR7-mediated activation of NF-κB and p38. The role of HRS in the production of proinflammatory cytokines, a downstream event of the signaling pathway, was then evaluated. *CSF3*, *IL-1β*, and *IL-6* mRNAs and proteins were induced in R848-stimulated or EV71-infected macrophages, but this induction was reduced in the presence of siR-HRS (Fig 9C and 9D), suggesting that HRS plays a critical role in the TLR7-induced production of proinflammatory cytokines.

The effect of HRS on activation of TLR7 signaling was also verified in mouse primary cells. Mouse primary bone marrow-derived macrophages (BMDMs) were infected with lentivirus encoding shR-HRS (Lenti-shHRS). *HRS* mRNA and protein were down-regulated significantly by Lenti-shHRS in BMDMs (S6A Fig). IL-1β and IL-6 expression were induced by R848, but this induction was reduced in cells transfected with Lenti-shHRS (S6B Fig). Intracellular cytokine staining showed that IL-6 protein expression was induced by R848, but not in the presence of Lenti-shHRS in CD14+ cells (S6C Fig), suggesting that HRS is important for TLR7-mediated activation of proinflammatory cytokines in mouse primary cells during the signaling initiation.

To confirm the function of HRS in TLR7-triggered inflammatory responses, we mimicked inflammation *in vivo*. Numbers of CD68^+^ cells were significantly increased in the spleen of mice treated with R848 (S6D Fig), indicating that an inflammatory response is induced in macrophages during maturation and activation as demonstrated previously [30]. Mice were tail vein injected with siR-HRS-m (a 5’-cholesterol-modified siRNA duplex targeted to HRS) and siR-NC-m (its control), followed by intraperitoneal injection with R848, and then PBMCs and splenocytes were isolated (Fig 9E). *HRS* and *IL-6* mRNA were induced by R848, but significantly reduced in the presence of siR-HRS-m in PBMCs and splenocytes (Fig 9F and 9G). Moreover, IL-1β, IL-6, TNFα and IL-10 protein expression was induced after R848 stimulation, but this induction was reduced by siR-HRS-m in sera of mice (Fig 9H). These results suggest that knock-down of HRS down-regulates TLR7-mediated production of proinflammatory cytokine. Taken together, HRS plays a vital role in proinflammatory cytokine production during TLR7-mediated inflammation *in vitro* and *in vivo* upon R848 stimulation.

### HRS is crucial for TLR7-induced production of IFNs during viral infection *in vitro* and *in vivo*

**Fig 10:**
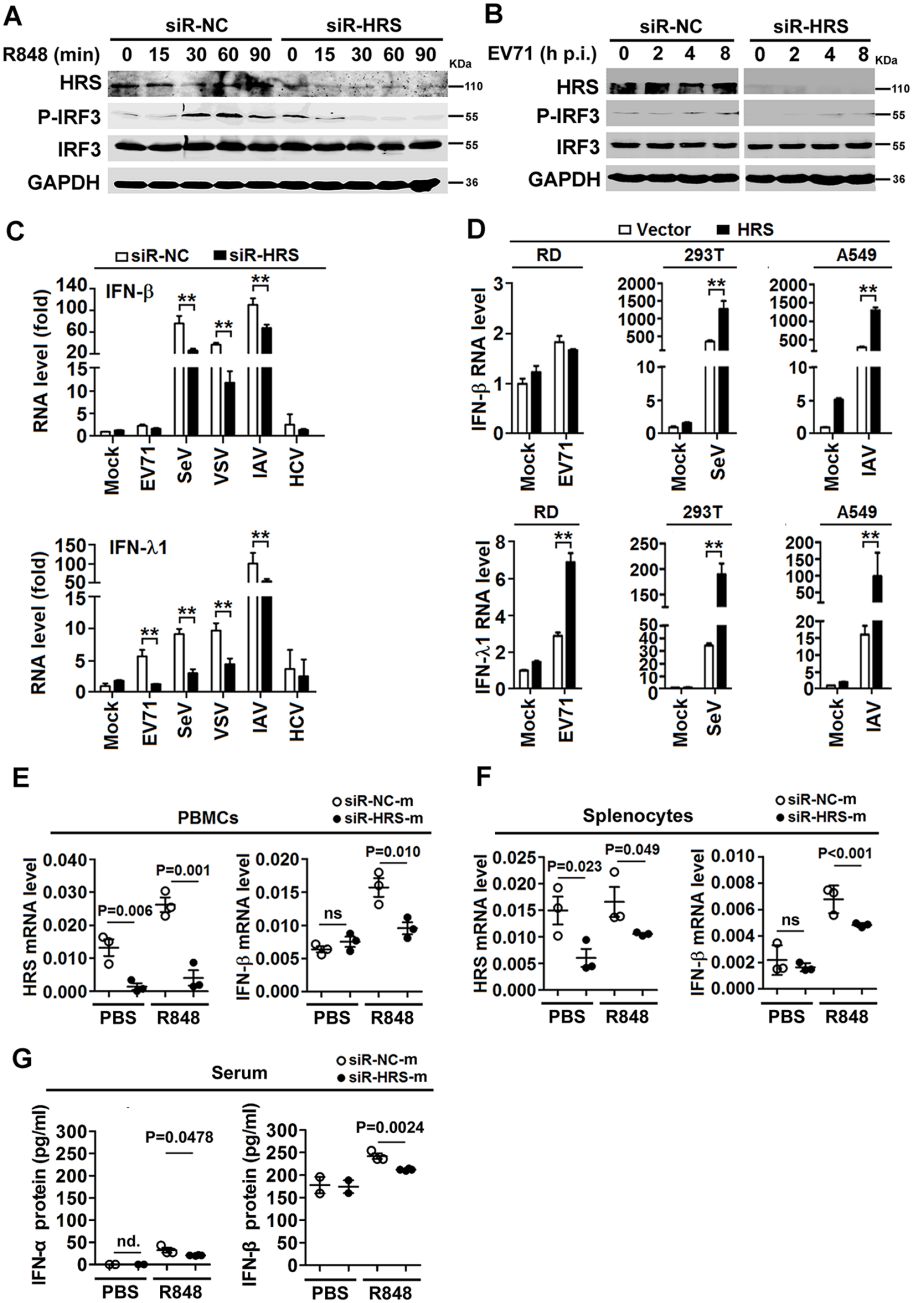
HRS promotes the production of interferons mediated by TLR7 signaling. (**A** and **B**) Macrophages were transfected with siR-HRS or siR-NC and then stimulated with R848 (**A**) or infected with EV71 (**B**) for various time periods as indicated. The proteins in the cell lysates were detected using Western blotting analyses with indicated antibodies. (**C**) Macrophages were transfected with siR-HRS or siR-NC and then stimulated with R848. *IFN-β* and *IFN-λ1* mRNA levels were determined using qPCR. (**D**) RD, 293T, and A549 cells were transfected with pcDNA3.1(-)-Myc or pcDNA3.1(-)-HRS-Myc and infected with EV71, SeV or IAV for 12 h, *IFN-β* and *IFN-λ1* mRNA levels were determined using qPCR. **, P<0.01. Graphs show means ± SD, n = 3. (**E**–**G**) PBMCs and splenocytes were isolated from the treated mice as in Fig 9E. IFN-β mRNA levels expressed in mouse PBMCs (**E**) and splenocytes (**F**) were determined using qPCR (each group, n = 3). IFNs proteins in mouse serum (**G**) were determined using ELISA (n = 2–4). ns, not significant; nd., not detected. Data show means ± SEM (standard error of the mean).

The role of HRS in the regulation of IFN signaling in response to virus infection was investigated. In R848-stimulated macrophages, phosphorylation of IRF3 was reduced in cells transfected with siR-HRS (Fig 10A). Similarly, in EV71-infected macrophages, phosphorylation of IRF3 was reduced by siR-HRS (Fig 10B, left *vs*. right). *IFN-β* mRNA expression was induced by SeV, vesicular stomatitis virus (VSV) and influenza A virus (IAV) infection, whereas the induction was abrogated in the presence of siR-HRS (Fig 10C, upper); *IFN-λ1* mRNA expression was activated by EV71, SeV, VSV, and IAV infection, whereas the induction was reduced by siR-HRS (Fig 10C, bottom). We also noticed that *IFN-β* mRNA was not induced by EV71 or HCV infection, and *IFN-λ1* mRNA was not induced by HCV infection. In addition, *IFN-β* was induced by SeV and IAV infection and it was further enhanced by HRS overexpression (Fig 10D, upper); while *IFN-λ1* was induced by EV71, SeV, and IAV infection and further facilitated by HRS overexpression (Fig 10D, bottom). These results demonstrate that HRS promotes TLR7-mediated IFN production during viral infection.

Moreover, *IFN-β* mRNA expression was induced by R848 and this was reduced by Lenti-shHRS in mouse BMDMs (S7A Fig), and IFN-β protein expression was enhanced by R848 and this enhancement was reduced by Lenti-shHRS in CD14+ cells (S7B Fig), indicating that HRS plays a role in TLR7-mediated activation of IFN-β in mouse primary cells. Furthermore, *HRS* and *IFN-β* mRNAs expression was enhanced by R848, but reduced in the presence of siR-HRS-m in mouse PBMCs (Fig 10E) and splenocytes (Fig 10F). IFN-α and IFN-β protein expression was induced by R848, but repressed in the presence of siR-HRS-m in mouse sera (Fig 10G). In conclusion, HRS plays an important role for TLR7 mediated IFN production in human macrophages and in mice.

## Discussion

Activation of TLRs initiates the integration of contextual cues and signals to regulate host inflammatory and immune responses [38]. TLR7 recognizes viral ssRNA to induce a wide range of proinflammatory cytokines and IFNs [39]. Here, we reveal a novel mechanism underlying the regulation of TLR7 signaling. Clinical investigation, animal study, and cellular analysis demonstrate that the productions of 3 important proinflammatory cytokines CSF3, IL-1β, and IL-6 are induced during EV71 infection. TLR7/NF-κB and TLR7/MAPK pathways are involved in such activation, which is consistent with a previous report showing that TLR7 activates MAPK and NF-κB signaling [40]. EV71 induces multiple TLRs accompanied with excessive production inflammatory factors. In intestinal epithelial cell, TLR7 mRNA is upregulated by EV71, suggesting that TLR7 mediates inflammatory response during EV71 infection [14, 15]. EV71 infection also stimulates TLR3 signaling in macrophage, leading to activation of iNKT (invariant natural killer T) cells [25]. In our study, we illustrate that EV71 infection facilitates TLR7 signaling in macrophages, resulting in induction of proinflammatory cytokines.

By using a stable cell line HEK293T/TLR7/NF-κB, we discovered that TLR7, MyD88, and IRAK1 are required for EV71-induced activation of NF-κB. Mini-library RNAi screening revealed that HRS is involved in TLR7/NF-κB-mediated inflammatory response. TLR7 and HRS are induced and highly correlated in EV71-infected patients, mice and cultured cells, which implicates that TLR7 and HRS shared similar function or had protein-protein interaction based on a co-expression prediction [41]. In the process of identifying HRS-interacting proteins, we reveal that HRS binds directly with TLR7 and TAB1, and co-localizes with TLR7 and TAB1 at foci in specific areas in the cytoplasm, indicating that HRS acts as a regulator or component of the TLR7 complex. HRS mediates endosome trafficking by binding with targeted proteins to deliver internalized cargos in the endocytic pathway [42]. We showed that HRS and TLR7 co-localize with clathrin at foci in the cytoplasm of infected cells. Because clathrin mediates endosomal trafficking events [31] and endosomes are the major sites for TLR complex assembly and antiviral response initiation [33, 43], we suggest that HRS regulates TLR7 signaling in the endosomes.

Upon stimulation, intracellular TLR7 moves from the ER to endosome *via* a clathrin-dependent endocytic pathway [44], a series of accessory molecules act as delivery cofactors, chaperones or trafficking proteins for biosynthesis and activation of TLR7 [45, 46]. Our results indicate that HRS is a key cofactor for TLR7 activation in endosomal location, in which HRS recruits TLR7 to facilitate it from early-endosome to late-endosome. We showed that the FYVE domain is required for HRS in the facilitation of TLR7 complex assembly from early- to late-endosome, which confirms that HRS enhances TLR7 signaling in the endosome. Thus, HRS regulates the TLR7 complex components in endosomes and may play a role in the initiation of immune response. In bone morphogenetic protein (BMP) signaling, HRS facilitates the crosstalk between SMADs and TAK1, which subsequently stabilizes the interaction between Smad2/Smad3 and TGF-β receptor and functions in internalization with the endocytic machinery in endosomes [47, 48]. We demonstrate that HRS acts as a key component in TLR7 signaling to facilitate the assembly of the TLR7/MyD88/IRAK1 complex by recruiting TLR7, and plays important roles in TLR7 signaling activation and initiation of the inflammatory response.

TLR7 stimulates signaling through TIR domain, triggers the binding of MyD88, activates IRAK1, and recruits TRAF6 and TBK1 [49]. Recruitment of the cellular factors activates multiple intracellular signaling cascades MAPK, NF-κB, and IRF3/7 to induce proinflammatory cytokines and IFNs [3]. We reveal that HRS is important for activation of NF-κB, IκBα, and p38 during viral infections, suggesting that HRS plays critical roles in TLR7-mediated activation of NF-κB, p38 MAPK, and IRF3 pathways. TLR7 in the endosomes and endolysosomes plays an important role in the initiation of antiviral responses [43]. We demonstrate that HRS is involved in TLR7-mediated induction of IL-1β and IL-6 in mice primary BMDMs and human cells, and stimulates cytokine production by facilitating the TLR7 complex assembly and NF-κB signaling.

**Fig 11:**
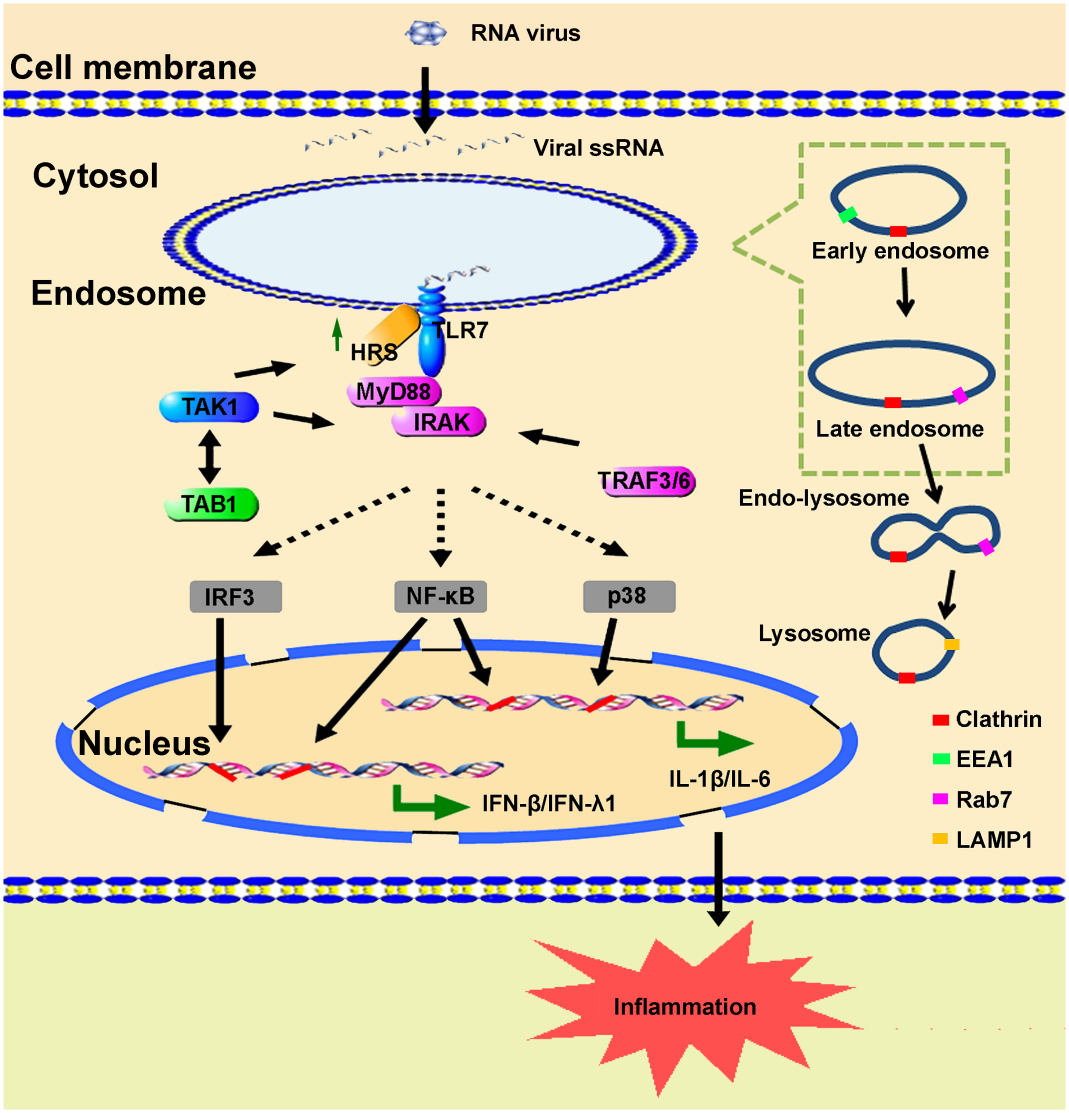
A proposed mechanism underlying HRS regulates TLR7-mediated inflammatory and immune responses. Upon infection, viral ssRNAs are recognized by TLR7 to trigger signaling events. TLR7 signaling is initiated by binding to MyD88 and IRAK and recruiting TRAF6, TAK1, and TAB1/2 to multiple intracellular signaling cascades, including p38 MAPK, NF-κB, and IRF3/7 to induce IFNs and proinflammatory cytokines production. This study reveals that ssRNA viruses induce HRS, which in turn induces immune and inflammatory responses by regulating TLR7 pathway. Initially, virus-induced HRS acts as a key component or regulator in TLR7 signaling to facilitate the assembly of the TLR7 complex by binding with TLR7 and TAB1. HRS further stimulates NF-κB and IRF3 pathway to induce IFNs, resulting in the induction of innate immune response. HRS also facilitates NF-κB and p38 pathway to induce proinflammatory cytokines, leading to the induction of inflammatory responses.

In summary, we reveal a novel mechanism underlying the regulation of TLR7 signaling (Fig 11). EV71 infection initially induces the production of HRS, which subsequently acts as a key regulator or component of the TLR7 complex coupling with endosomal location by binding with TLR7 and TAB1 to facilitate the assembly of the TLR7 complex. On one hand, HRS activates TLR7/NF-κB/IRF3 signaling to promote the production of IFNs, resulting in induction of innate immune response. On the other hand, HRS simulates TLR7/NF-κB/p38 signaling to enhance production of proinflammatory cytokines, leading to the induction of inflammatory response. Therefore, HRS-mediated TLR7 complex assembly may provide an important mechanism for the regulation of host immune and inflammatory responses during viral infection.

## Materials and methods

### Clinical analyses

The cases of mild HFMD were defined as patients with vesicular lesions on their palms, feet, and mouth, with or without fever, and severe HFMD were accompanied by neurologic or cardiopulmonary complications. All participants were diagnosed with EV71 infection by presence of EV71 RNA with specific EV71 *VP1* primer and confirmed as negative for other enterovirus. Peripheral blood specimens were obtained from 40 EV71-infected patients hospitalized at the Wuhan Medical Treatment Center and Wuhan Infectious Diseases Hospital from May 2, 2016 to May 10, 2017. The peripheral blood samples were obtained from all subjects on admission from 12 to 24 h. The blood samples were immersed in ice and transported immediately to the laboratory for processing. Matched by age and sex, peripheral blood specimens were collected from 36 EV71-negative healthy individuals in a local blood donation center as controls. All individuals did not suffer any concomitant disease at the moment of sampling, did not show any serological markers suggestive of autoimmune disease, and had not received any antiviral or immunomodulatory therapy prior to this study.

### Animal studies

BALB/c mice were purchased from Shanghai Laboratory Animal Center. The mice were housed under specific pathogen-free conditions in individually ventilated cages. One-day-old suckling mice were intraperitoneally (i.p.) mock-infected or infected with 1×10^7^ plaque-forming units (PFU) of MA-EV71 in 50 μl PBS. Following EV71 infection, the mice were scored as follows: 0, healthy; 1, ruffled hair and hunchbacked; 2, limb weakness; 3, paralysis in one limb; 4, paralysis in both limbs; and 5, death. For EV71 infection mice model, mice were sacrificed at 10 days post-infection.

In siRNA *in vivo* transfection experiments, 5’-cholesterol-modified siRNA duplexes (siR-NC: 5’-TTCTCCGAACGTGTCACGT; siR-HRS-mouse: 5’-CGCAUGAAGAGCAACCACA; GenePharma, Shanghai, China) were diluted in 0.9% NaCl solution and injected into a mouse (weight = 20 g) *via* the tail vein (injection volume = 100 μl) based on a final dose of 2 mg/kg twice for 2 d. Subsequently, R848 (a TLR7 agonist) diluted in PBS was injected intraperitoneally into a mouse at a final dose of 2 mg/kg. Two days after the final injection, mice were sacrificed and spleen cells and PBMCs were isolated for analyses. The spleen tissues were fixed in 3.7% paraformaldehyde and then applied to immunohistochemistry (IHC) or immunofluorescence (IF) staining.

### Ethics statement

The study was conducted according to the principles of the Declaration of Helsinki and approved by the Institutional Review Board of the College of Life Sciences, Wuhan University, in accordance with its guidelines for the protection of human subjects. All participants have provided written informed consent to participate in the study.

All animal studies were performed in accordance with the principles described by the Animal Welfare Act and the National Institutes of Health guidelines for the care and use of laboratory animals in biomedical research. All procedures involving mice and experimental protocols were approved by the Institutional Animal Care and Use Committee (IACUC) of the College of Life Sciences, Wuhan University (Permit numbers: 2015–006).

### Viruses and infections

The growth, virus titration and inoculation of enterovirus 71 (EV71) [50, 51], Sendai virus (SeV) [52], vesicular stomatitis virus (VSV), influenza A virus (IAV) [53], and hepatitis C virus (HCV) [54] were performed as described previously.

EV71 virus strain (Xiangyang-Hubei-09) was isolated previously in our laboratory (GenBank accession no. JN230523.1). Virus stock was propagated in RD cells. Indiana serotypes of VSV strain were provided by the China Center Type Culture Collection. IAV strain A/HongKong/498/97 (H3N2) was provided by the China Center for Type Culture Collection. The virus stock was propagated in human lung epithelial cells (A549) were cultured in F12K medium (Invitrogen). HCV genotype 2a strain JFH-1 was kindly provided by Takaji Wakita. Huh7.5.1 cells were infected with JFH-1 at a multiplicity of infection (MOI) of between 0.1 and 5. HCV was propagated for 6 days before collection. Virus stock was obtained after filtering of the cell supernatant. Viral titers were quantified using a commercial kit (HCV RNA qPCR diagnostic kit; KHB Company, Shanghai, China). Aliquots were stored at -80°C prior to use. Cells were infected with virus at the indicated multiplicities of infection (MOI) and unbound virus was washed away 2 h later, and then incubated at 37°C for an additional 10 h.

### Cell lines and cultures

Human leukemic monocytes (THP-1) cells, human embryonic kidney 293T (HEK 293T) cells, human lung adenocarcinoma A549 (A549) cells, human hepatoma (Huh7.5.1) cells, and mouse Raw264.7 cells were purchased from American Type Culture Collection (ATCC) (Manassas, VA, USA). Human embryonal rhabdomyosarcoma (RD) cells and HeLa cells were obtained from China Center for Type Culture Collection (CCTCC) (Wuhan, China).

THP-1 cells were cultured in RPMI 1640 medium (Invitrogen, Carlsbad, CA), supplemented with 10% fetal bovine serum (FBS), 100 U/ml penicillin and 100 mg/ml streptomycin sulfate at 37°C in a 5% CO_2_ incubator. Macrophages were derived from THP-1 cells induced by 100 nM 12-O-tetradecanoyl-phorbol-13-acetate (TPA) (Sigma, St. Louis, MO) for 72 h. Cell surface markers for differentiated macrophages were detected by flow cytometry. PBMCs were obtained by density centrifugation of peripheral blood samples diluted 1:1 in pyrogen-free saline over Histopaque (Haoyang Biotech, Beijing, China). Cells were washed twice in saline and suspended in culture medium.

HEK293T/TLR7/NF-κB stable cell lines were generated after co-transfecting HEK293T cells with TLR7 expression plasmid pTLR7 and 5 × NF-κB luciferase reporter construct.

For the culture of mouse BMDMs, male C57BL/6 TLR7^-/-^ mice (a kind gift from Dr. Ling Zhao, Huazhong Agricultural University, Wuhan, China) 4–6 weeks of age were sacrificed and pelvic and femoral bones collected to separate bone marrow. Cells were cultured in RPMI 1640 medium with 10% fetal bovine serum (FBS) following induction with 100 ng/ml mouse granulocyte-macrophage colony-stimulating factor (GM-CSF) (Peprotech, NJ, USA) for 3 d until cells became attached BMDMs.

### Antibodies

Rabbit antibodies against TLR7, MyD88, NF-κB p65, and phosphorylated IκBα (p-IκBα, Ser32) were purchased from Abcam (Cambridge, United Kingdom). Mouse antibodies against HRS, IRAK1, and c-Myc Tag, goat antibodies to TAB1, and normal rabbit IgG were purchased from Santa Cruz Biotechnology (Santa Cruz, CA). Rabbit antibodies against phosphorylated NF-κB p65 (p-p65, Ser536), phosphorylated p38 MAPK (p-p38, Thr180/Tyr182), p38 MAPK, Clathrin, Calnexin, EEA1, Rab5, Rab7, Rab11, LAMP1, Rcas1, and TRAF6 were purchased from Cell Signaling Technology (Beverly, MA). Rabbit antibodies against GFP, PKCζ, β-actin, and GAPDH were purchased from ProteinTech Group (Chicago, IL). Mouse antibody to EV71 VP1 was from Abnova Company (Taipei). Rabbit antibody to EV71 3C was raised against residues 76–88 of 3C protein (Abgent, Suzhou, China). Rat antibodies to FLAG, and anti-mouse CD68 were purchased from BioLegend (San Diego, CA).

### Reagents

The specific small interfering RNA (siRNA) to the negative control (NC), *HRS*, and mini-siRNA library were synthesized by RiboBio (Guangzhou, China). siR-NC targeting at sequence: 5’-TTCTCCGAACGTGTCACGT; siR-HRS targeting at sequence: #1: 5’-AGGTAAACGTCCGTAACAA; #2: 5’-GCATGAAGAGTAACCACAT.

All kinase inhibitors were purchased from Sigma Chemical Company (St. Louis, MO) and dissolved in DMSO upon use as described previously [54]. R848 (InvivoGen) dissolved in ddH_2_O and used at a final concentration of 100 ng/ml.

### Plasmids constructions

The full-length TLR7 gene (GenBank accession no. NM_016562) was amplified from THP-1 cells and the cDNA was inserted into the pCMV-Flag2B vector (Invitrogen) to create pFlag-TLR7, which encoded a Flag-TLR7 fusion protein. The plasmid pFlag-TLR7 (Y892A) expressing a mutant Flag-TLR7 (Y892A) fusion protein was constructed as described previously [21].

The full-length and truncated HRS genes (GenBank accession no. NM_004712) were cloned into pcDNA3.1(-)-Myc-His vector (Invitrogen) to generate plasmids encoding for HRS-Myc and dFYVE-HRS-Myc (with a deletion of amino acids 166–215).

The DNA fragments of TLR7, MyD88, IRAK1, TRAF3, TRAF6, TAB1, TAK1, and TBK1 genes were ligated into eGFP-C1 (Takara) vector to generate plasmids expressing GFP-tagged TLR7, MyD88, IRAK1, TRAF3, TRAF6, TAB1, TAK1, and TBK1 proteins, respectively.

The shTLR7 plasmid expressing a short-hairpin RNA (shRNA) (5’-GCCAACAACCGGCTTGATTTA) targeting TLR7 was generated by inserting the DNA fragment into the pSilencer 2.1-U6 neo vector (Ambion, Inc., Austin, TX). shGFP, shMyD88, shIRAK1, shp65, and shp50 were constructed as described previously according to targeting sequences of GFP, MyD88, IRAK1, p65, and p50, respectively [52].

The primers used in this study are listed in S3 Table.

### Transfection, reporter assays, RNAi experiments, and quantitative PCR

HEK 293T cells (1×10^5^ cells) were co-transfected with pFlag-TLR7 or vector (0.24 μg) and NF-κB luciferase reporter (0.08 μg) using Lipofectamine 2000 (Invitrogen) in each well of 24-well plate. The determination of NF-κB reporter luciferase activity was performed as described previously [54]. HEK 293T cells (1×10^5^ cells) were co-transfected with the luciferase reporter plasmid (80 ng), pRL-TK Renilla luciferase control plasmid (40 ng), and the indicated siRNA (final concentration, 20 nM). After 24 h, luciferase activities were determined with the Dual-Luciferase Reporter Assay System (Promega) according to the manufacturer’s instructions. Data were normalized for transfection efficiency by dividing firefly luciferase activity with that of Renilla luciferase.

For knock-down HRS, the specific HRS siRNA or control siRNA were transfected into THP-1 cells using INTERFERin (Polyplus Transfection Company, Illkirch, France). Cells were treated with R848 or EV71 at 36 h post-transfection, and total cellular RNA was extracted using TRIzol reagent (Invitrogen).

Reverse transcription and quantitative PCR (qPCR) was performed as described previously [54]. Quantitative RT-PCR (qPCR) analysis was performed using the Roche LightCycler 480 and SYBR RT-PCR kits (Roche); each 20 μl reaction contained 0.5 μM of each PCR primer, 10 μl of SYBR Green PCR master mix, 1 μl of diluted template, and RNase-free water. The data represent absolute mRNA copy numbers normalized to GAPDH used as a reference gene. Relative fold expression values were determined by using the ^ΔΔ^Ct method. The information of real-time PCR primers is listed in Table 2.

**Table 2:** List of primers used for qPCR in this study.

Primer title	Orientation
qRT GAPDH F	5’-AAGGCTGTGGGCAAGG-3’
qRT GAPDH R	5’-TGGAGGAGTGGGTGTCG-3’
qRT TLR3 F	5’-CCTGGTTTGTTAATTGGATTAACGA-3’
qRT TLR3 R	5’-TGAGGTGGAGTGTTGCAAAGG-3’
qRT TLR4 F	5’-CAGAGTTTCCTGCAATGGATCA-3’
qRT TLR4 R	5’-GCTTATCTGAAGGTGTTGCACAT-3’
qRT TLR7 F	5’-TTTACCTGGATGGAAACCAGCTA-3’
qRT TLR7 R	5’-TCAAGGCCTGAGAAGCTGTAAGCTA-3’
qRT TLR9 F	5’-GGACCTCTGGTACTGCTTCCA-3’
qRT TLR9 R	5’-AAGCTCGTTGTACACCCAGTCT-3’
qRT IL-8 F	5’-AAGGAACCATCTCACTGTGTGTAAAC-3’
qRT IL-8 R	5’-TTAGCACTCCTTGGCAAAACTG-3’
qRT CXCL-10 F	5’-GTGGCATTCAAGGAGTACCTC-3’
qRT CXCL-10 R	5’-TGATGGCCTTCGATTCTGGATT-3’
qRT TNFα F	5’-TCTCGAACCCCGAGTGACA-3’
qRT TNFα R	5’-GGCCGGCGGTTCA-3’
qRT IL-12 F	5’-TCTCGGCAGGTGGAAGTCA-3’
qRT IL-12 R	5’-ACTTTGGCTGAGGTTTGGTCTG-3’
qRT HRS F	5’-ACCGGAACTACTGGGAGAAGAAGCA-3’
qRT HRS R	5’-TCCTCAGACTCGCCATTGTGGAAC-3’
qRT CSF3 F	5’-ATAGCGGCCTTTTCCTCTACC-3’
qRT CSF3 R	5’-GCCATTCCCAGTTCTTCCAT-3’
qRT IL-1β F	5’-AACCTGCTGGTGTGTGACGTTC-3’
qRT IL-1β R	5’-AGCACGAGGCTTTTTTGTTGT-3’
qRT IL-6 F	5’-GTACATCCTCGACGGCATCTCA-3’
qRT IL-6 R	5’-GCACAGCTCTGGCTTGTTCCTC-3’
qRT IFN-β F	5’-GAGGCTTGAATACTGCCTCAA-3’
qRT IFN-β R	5’-TCCTTGGCCTTCAGGTAATGCAGA-3’
qRT IFN-λ1 F	5’-CTTCCAAGCCCACCACAACT-3’
qRT IFN-λ1 R	5’-GGCCTCCAGGACCTTCAGC-3’
qRT TLR7 (mouse) F	5’-GTTCTATGGAGAGCCGGTGATA-3’
qRT TLR7 (mouse) R	5’-ATTCTTTAGATTTGGCGGCATA-3’
qRT HRS (mouse) F	5’-ACCGGAACTACTGGGAGAAGAAGCA-3’
qRT HRS (mouse) R	5’-CATTCTGGTACTGCTCACTAAAGGG-3’
qRT CSF3 (mouse) F	5’-ATGCCGAAGGCTTCCCTG-3’
qRT CSF3 (mouse) R	5’-AGGAGACCTTGGTAGAGG-3’
qRT IL-1β (mouse) F	5’-AGCTTCAGGCAGGCAGTATC-3’
qRT IL-1β (mouse) R	5’-CGTCACACACCAGCAGGTTA-3’
qRT IL-6 (mouse) F	5’-AGACAAAGCCAGAGTCCTTCAGAGA-3’
qRT IL-6 (mouse) R	5’-GCCACTCCTTCTGTGACTCCAGC-3’
qRT IFN-β (mouse) F	5’-GGATCCTCCACGCTGCGTTCC-3’
qRT IFN-β (mouse) R	5’-CCGCCCTGTAGGTGAGGTTGA-3’
qRT GAPDH (mouse) F	5’-ATGTTTGTGATGGGTGTGAA-3’
qRT GAPDH (mouse) R	5’-ATGCCAAAGTTGTCATGGAT-3’

The sequences of primer involved in this study are list in Table. F, forward; R, reverse; qRT, the primers used in quantitative real-time PCR. The mouse genes are indicated in brackets.

### Enzyme-linked immunosorbent assay (ELISA)

The tested proteins in the sera of clinical samples were measured with Human IL-1β immunoassay (BD Biosciences, CA) and Human CSF3 and IL-6 immunoassay (R & D systems, Minneapolis, MN). Proteins in mice sera were measured with Mouse CSF3, IL-6, IL-1β, IL-10 and TNFα immunoassay (4A Biotech, Beijing, China), as well as IFN-α and IFN-β immunoassay (eBioscience; Santa Diego, CA) following the manufacturer’s instructions, respectively.

The 30 cytokines ELISA assay for EV71-infected THP-1 supernatant was described in details as follow. THP-1 cells (1×10^6^ per well) were distributed into 6-well culture plates with 10% FBS, and then incubation with EV71 at an MOI = 5 for 2 h at 37°C. Suspended cells were centrifuged at 1000 *g* for 10 min. Cells were resuspended in PBS to wash away the unbound virus. After three times washing in PBS, cells were cultured in 2 ml of FBS-free RPMI 1640 for additional 12 h. Finally, the cell supernatant was harvested after the centrifugation at 10000 *g* for 5 min for ELISA assay. 30 cytokines in cell supernatants were measured with indicated antibodies-coated plate of Human Cytokine ELISA Plate Assay I kit (Signosis, Sunnyvale, CA, USA). The simultaneous quantification of each cytokines concentration in cell supernatants was determined according to absorbance value. Each cytokine induction was normalized to mock control and data are shown as fold changes in a heatmap visualization.

### Immunoprecipitation and immunoblot analysis

Human THP-1 cells (5×10^6^ cells) were cultured in 10 cm dish and harvested after R848 stimulation as indicated time points and lysed in 1 ml RIPA buffer (50 mmol/l Tris-HCl pH7.4, 150 mmol/l NaCl, 1% sodium deoxycholate, 0.5 mol/l EDTA, 1 mmol/l NaF, 1% Nonidet P-40, supplemented by 10% proteinase inhibitors cocktail). 1/10 lysate (100 μl) was reserved for direct immunoblot analysis while the rest was successively incubated with rabbit anti-TLR7 or normal rabbit IgG antibodies for 4 h and with protein G agarose (GE Healthcare) for another 1 h. After 5 rounds of washes, proteins were fractionated by SDS-PAGE and transferred to nitrocellulose membrane (Amersham, Piscataway, NY). Nonspecific sites were blocked with 5% nonfat dried milk (BD Biosciences, San Jose, CA) for 1 h at room temperature. After 3 times of PBS wash, the nitrocellulose membrane was incubated with primary and secondary antibodies. Blots were analyzed using a Luminescent Image Analyzer (Fujifilm LAS-4000).

### GST pull-down assays

The expression and purification of GST or GST-HRS proteins were performed as follows. The cDNA encoding HRS was cloned into pGEX6p-1 vector (GE Healthcare) and transformed to *E*. *coli* BL21 strain. A single colony was grown in 5 ml LB culture containing 100 μg/ml of Ampicillin overnight. 1 ml of bacteria culture was then transferred to a 50 ml flask with 20 ml of LB and 100 μg/ml of Ampicillin in an orbital shaker at 37°C, 200 rpm for 2 h until A_600_ = 0.5–0.6. IPTG was incubated with the culture at a final concentration of 0.6 mM at 37°C, 200 rpm for additional 6 h. Cells were centrifuged at 8,000 g for 10 min at 4°C and the supernatant was removed. The pellets were resuspended with 10 ml PBS containing 0.1 mM PMSF and lysed with the ultrasonic processor. The lysate was centrifuged at 8,000 g for 10 min at 4°C and the supernatant was prepared. Before the affinity of the GST-fusion to the column, 2 ml of glutathione-sepharose beads (GE Healthcare) was equilibrated with 10 volume of lysis buffer (50 mM Tris-HCl pH8.0, 150 mM NaCl, 1 mM DTT, 0.1 mM EDTA and 0.1 mM PMSF) at 4°C. Unbound lysis was washed away with 15 ml wash buffer (50 mM Tris-HCl pH8.0, 150 mM NaCl, 1 mM DTT). Finally, GST-fusion protein was eluted with 5 ml of elution buffer (125 mM Tris-HCl pH8.0, 45 mM NaCl, 0.0001% Triton X-100, 5 mM EDTA and 30 mM reduced Glutathione).

The cell lysate was incubated with 50 μl of glutathione-sepharose beads (50/50 slurry in lysis buffer) and 25 μg of GST or GST-HRS for 2 h at 4°C with end-over-end mixing. The beads were washed four times with 1 ml of ice-cold lysis buffer. After discarding the last wash, proteins bound to the probe protein were dissociated for immunoblot analysis.

### Immunofluorescence microscopy

Immunofluorescence microscopy experiments were performed as described previously [50]. Briefly, macrophages were seeded on 20-mm cover slips and then treated with R848 for 30 min, or infected with EV71 or SeV for 4 h. Then, the culture medium was removed and cells were washed with PBS, fixed with 3.7% formaldehyde for 20 min, and permeabilized using 0.4% Triton X-100 for 5 min at room temperature. After another PBS wash, the cells were blocked in PBS containing 5% BSA for 1 h, incubated with antibodies for 3 h at 37°C. The samples were incubated with FITC-conjugated donkey anti-rabbit immunoglobulin G (IgG) and Cy3-conjugated donkey anti-mouse IgG (ProteinTech Group) for 45 min at room temperature. To stain nuclei, 1 mg/ml DAPI (Roche) methanol solution was added and samples incubated for 15 min at room temperature. After washing with PBS, samples were visualized by confocal laser scanning microscopy (Fluoview FV1000; Olympus, Tokyo, Japan).

### Bimolecular fluorescence complementation (BiFC) assays

The N-terminal truncated (VN-173) and the C-terminal truncated (VC-155) version of nonfluorescent Venus YFP fragments vectors were brought here (Addgene plasmid # 22010) [55]. Human TLR7 and TAB1 cDNA were subcloned in VN-173, and human HRS cDNA was ligated to VC-155 without their stop codons using ClonExpress MultiS One Step Cloning Kit (Vazyme Biotech Co., Ltd, Nanjing, China), respectively. The resulting plasmids or empty vectors were cotransfected into HEK293T cells using Lipofectamine 2000. At 24 h post-transfection, cells were pre-cultured at 4°C for 10 min. The fusion proteins in living cells were observed by confocal microscopy.

### Subcellular fractionation experiments

Macrophages were treated with or without R848 (100 ng/ml) for 30 min. The harvested cells were washed extensively with PBS. The pellet obtained following centrifugation of cells for 5 min at 2,000 *g* was washed twice with TS buffer (0.25 M sucrose, 10 mM Tris-Cl, pH 7.4) then were homogenized with 40 strokes in a Douncer on ice, followed by centrifugation at 1,000 *g* for 20 min at 4°C. To separate the membrane pellets from the cytosol, the supernatant fractions were subjected to further centrifugation at 100,000 *g*. To separate subcellular fractionation, the supernatant was adjusted by adding 1.2 volume of 62% sucrose, sequentially overlaid with 1.5 volume of 35% sucrose, 1 volume of 25% sucrose and filled up with TS buffer to the rest of the tube. Sucrose gradient centrifugation was performed in a Beckman SW41 Ti rotor at 210,000 *g* at 4°C for 2 h. The fractions were collected from the top to bottom by subsequent detection in the distribution of intracellular markers by SDS-PAGE and Western blotting.

### Lentivirus package

Recombinant lentivirus carrying shRNA targeting HRS (Lenti-shHRS-1: 5'-AAAGGTAAACGTCCGTAACAA, and Lenti-shHRS-2: 5’- CCGCATGAAGAGTAACCACAT) or the control (Lenti-shNC: 5’-CAACAAGATGAAGAGCACCAA) were constructed from plasmids psPAX2, pMD2.G and pLKO.1 (Addgene, Cambridge, MA) and generated from co-transfected HEK293T cells.

### Cell viability assay

Cells were seeded in 96-well plate after treatment. Cell viability was determined by CellTiter 96 AQueous One Solution Cell Proliferation Assay (Promega) according to the instructions provided by the manufacturer.

### Flow cytometry

Flow cytometric measurements were performed using a Beckman Coulter flow cytometry (Fullerton, CA). For detection of cell surface markers, Fc receptors were blocked by incubating 100 mg recombinant human IgG (Sigma-Aldrich) with cells for 15 min at 4°C prior to antibody staining. 1 mg of monoclonal PE mouse IgG1κ anti-human CD14 antibody, or the relevant PE mouse IgG1κ Isotype Ctrl (FC) antibody (BioLegend) was incubated with samples containing 2 × 10^5^ cells for 15 min at 4°C. Following incubation samples were washed and resuspended in phosphate buffered saline (PBS) and 10,000 events recorded. Data was analyzed using Summit software, version 5.0.1 (Beckman Coulter Inc.). PE anti-human CD14 or isotype, PE/Cy7 anti-mouse CD14 or isotype control, PE anti-mouse IL-6 or isotype control were purchased from BioLegend (San Diego, CA). FITC anti-mouse IFN-β or isotype control antibodies were from R&D systems (Minneapolis, MN).

For intracellular cytokine staining, BD GolgiPlus^™^ (1 μg/ml) and BD GolgiStop (0.7 μg/ml) protein transport inhibitor (BD Biosciences, CA) was added for 5 h during antigen stimulation. Intracellular cytokines were stained with indicated antibodies with the Cytofix/Cytoperm Kit (BD Biosciences). Cells were acquired on a FACS Calibur flow cytometer (BD Biosciences), and data were analyzed with FlowJo software (Tree Star, Inc, Ashland, OR).

### Statistics

All experiments were reproducible and each set was repeated at least three times with similar results. All data were recorded as means ± standard deviation (SD) unless stated otherwise. Statistical testing was performed using Prism 5 software (GraphPad Software Inc.) with the statistical tests indicated in the figure legends. A *P*-value < 0.05 was considered to indicate statistical significance.

## Supporting information

S1 Fig EV71 infection facilitates the induction of inflammatory cytokines. (**A**) PMBCs were isolated from EV71-infected or uninfected persons. Total RNA was extracted, followed by semi-RT PCR with specific EV71 VP1 and GAPDH (an internal control) primers. The results represent the EV71 RNA detection in all collected samples. (**B** and **C**) EV71 VP1 viral RNA (**B**) and protein (**C**) expressed in PBMCs and spleen of EV71-infected or non-infected (Mock) mice (each group, n = 7) were measured using qPCR and IHC, respectively. (**D**) Macrophages were differentiating from human THP-1 cells by the treatment of 100 nM TPA. CD14 was selected as a cell surface marker of macrophage derived from THP-1 cells. The surface marker of differentiated macrophages was detected by flow cytometry. Histograms of median fluorescent intensity of CD14 of THP-1 cells untreated (-TPA) or treated with TPA (+TPA). Data are representative of three independent experiments.
(TIF)

S2 Fig EV71 induces inflammatory cytokines by activating TLR7 signaling. (**A**) THP-1 cells were treated with individual kinase inhibitors for 6 h, as indicated. Cell viabilities were then determined. (**B**) THP-1 cells were treated with individual kinase inhibitors for 6 h, as indicated, and infected with EV71 for 12 h. EV71 3C and β-actin proteins were detected by Western blotting analyses. (**C** and **D**) Mouse Raw264.7 cells were treated with indicated kinase inhibitors for 6 h and infected with EV71 (MOI = 5) for 24 h. *CSF3*, *IL-1β*, and *IL-6* mRNAs **(C)** and supernatants CSF3, IL-1β, and IL-6 proteins **(D)** were measured by qPCR and ELISA, respectively. (**E**) THP-1 cells were transfected with shTLR7 or shGFP, and selected with 300 μg/ml G418. TLR7 and β-actin proteins expressed in the cells were detected by Western blotting analyses using specific antibodies to the proteins. (**F**) Mouse bone marrow-derived macrophages (BMDM) isolated from TLR7 wild-type (WT) or TLR7 knock-out (TLR7^-/-^) mice were infected with EV71 (MOI = 5) for 24 h. The mouse CSF3, IL-1β, and IL-6 proteins in cell supernatants were measured by ELISA. (**G**) HEK293T cells were transfected with pFlag-TLR7, pFlag-TLR7(Y892A) (a mutant of TLR7), or the vector. TLR7 and β-actin proteins expressed in the cells were detected by Western blotting analyses using specific antibodies to the proteins. Data are shown as mean ± SD and correspond to a representative experiment out of three performed. ns, non-significant; *, *P* < 0.05; **, *P* < 0.01.
(TIF)

S3 Fig The assessment and integration of the protein-protein interaction networks of cellular factors in TLR7 signaling pathway. (**A**) Identified TLR7 signaling pathway associated factors and unknown or predicted proteins are integrated into available STRING database using version 10.0 of STRING software (http://string-db.org). Total selected 28 items represent in a form of node and the lines in different colors stand for the known or predicted interactions in TLR7 signaling pathway. (**B**) Stable HEK293T/TLR7/NF-κB reporter cells were transfected with plasmids encoding siRNAs specific to indicated genes and stimulated with R848. NF-κB activities were determined by luciferase activity assays. (**C**) THP-1 cells were transiently transfected with siRNA to HRS (siR-HRS) or its negative control (siR-NC) for 36 h. HRS and β-actin proteins were detected by Western blotting analyses. (**D**) THP-1 cells were transfected with siR-HRS or siR-NC for 24, 36, and 48 h. The cell viabilities were determined. (**E**) THP-1 cells were transfected with siR-HRS or siR-NC, and treated with Annexin V: FITC. The cell apoptosis was analyzed by Apoptosis Detection Kit (BD Biosciences, San Jose, CA).
(TIF)

S4 Fig HRS expression is upregulated through TLR7-mediated NF-κB signaling. (**A**) Bioinformatic prediction of NF-κB subunit binding sites in human and mouse *HRS* or *TLR7* promoter using P-Match 1.0 Public software (http://gene-regulation.com/). (**B**) Mouse Raw264.7 cells were treated with indicated kinase inhibitors for 6 h, and infected with EV71 (MOI = 5) for 24 h. (**C**) Mouse bone marrow-derived macrophages (BMDM) isolated from TLR7 WT or TLR7^-/-^ mice were infected with EV71 (MOI = 5) for 24 h. (**B** and **C**) The proteins expressed in the treated cells were detected by Western blotting. The indicated band intensity represents as fold changes to internal control by using Image J software analysis.
(TIF)

S5 Fig The immunohistochemistry (IHC) staining in the mice spleens. (**A**) Mice spleens from WT or TLR7^-/-^ mice were subjected to immunohistochemistry (IHC) staining with TLR7 antibody. Bar = 100 μm. (**B**) Mice were mock-infected or infected with EV71 and sacrificed at indicated period. Mice spleens were subjected to immunohistochemistry (IHC) staining with the anti-mouse CD68 antibody. Bar = 50 μm.
(TIF)

S6 Fig HRS activates cytokine production mediated by TLR7 signaling in mouse primary cells. (**A**) Mouse Bone marrow-derived macrophages (BMDMs) isolated from mice were infected with lentivirus coding siRNA to HRS (Lenti-siR-HRS-1 and -2) or the control (Lenti-siR-NC) for 72 h. The efficiency of knock-down of HRS is evaluated by the determination of HRS mRNA and HRS protein using qPCR (upper panel) and Western blotting analyses (lower panel). (**B** and **C**) BMDMs isolated from mice were infected with lentivirus coding siRNA to HRS or the control for 72 h and stimulated with or without R848 (100 ng/ml) for 12 h. IL-1β and IL-6 mRNA levels were determined using qPCR (**B**). IL-6 protein levels were determined using flow cytometry (**C**). (**D**) Mice were stimulated without or with R848 and sacrificed at indicated period. Mice spleens were subjected to immunohistochemistry (IHC) staining with the anti-mouse CD68 antibody. Bar = 50 μm. Results were expressed as fold induction relative to control. **, *P* < 0.01.
(TIF)

S7 Fig HRS activates IFN-β production mediated by TLR7 signaling in mouse primary cells. (**A**) Macrophages were transfected with siR-NC or siR-HRS and stimulated with R848. The level of IFN-β mRNA was determined using qPCR. (**B**) BMDMs isolated from mice were infected with lentivirus coding siRNA to HRS or the control for 72 h and stimulated with or without R848 (100 ng/ml) for 12 h. IFN-β protein level was determined using flow cytometry.
(TIF)

S1 Table Analysis of the effects of EV71 infection on human cytokines and chemokines expression by Human Cytokine ELISA Plate Array. THP-1 derived macrophages were infected with EV71 (at MOI = 5) or inoculated with UV-inactivated EV71 for 12 h. Levels of cytokines in cell supernatants were measured by a commercial kit (Human Cytokine ELISA Plate Assay I for Profiling 30 Cytokines kit, Signosis, Sunnyvale, CA, USA) according to manufacturers’ instructions. Data are shown as fold changes of protein expression in cell supernatants compared to mock samples. At least 2 times changes were considered statistically significant. *TNF-α, tumor necrosis factor alpha; IFN-γ, interferon gamma; CSF3, colony stimulating factor 3; GM-CSF, granulocyte-macrophage colony stimulating factor; IL-1α, interleukin 1 alpha; VEGF, vascular endothelial growth factor; EGF, epidermal growth factor; IL-6, interleukin 6; Resistin, also called RSTN or RETN1; PAI-1, plasminogen activator inhibitor type 1; IL-12, interleukin 12; IL-13, interleukin 13; Eotaxin-3, also named chemokine (C-C motif) ligand 26 (CCL26); PDGF-BB, platelet-derived growth factor beta polypeptide; PIGF-1, placental growth factor 1; β-NGF, nerve growth factor-beta; SCF, skp-cullin-F-box; MCP-1, monocyte chemotactic protein 1; MIP-1α, macrophage inflammatory protein 1 alpha; IL-2, interleukin 2; IL-4, interleukin 4; IL-8, interleukin 8; IL-10, interleukin 10; bFGF, basic fibroblast growth factor; Leptin, also called LEPD; IGF-1, insulin-like growth factor 1; TGF-β, transforming growth factor beta; Adipo, adiponectin; IL-17α, interleukin 17 alpha; IL-1β, interleukin 1 beta.
(DOC)

S2 Table List of genes information in RNAi screening used in this study. UQCRC1, ubiquinol-cytochrome C reductase core protein I; CTSW, cathepsin W; TRAF5, tumor necrosis factor receptor-associated factor 5; HERPUD1, homocysteine-inducible, endoplasmic reticulum stress-inducible, ubiquitin-like domain member 1; FBXL7, F-box and leucine-rich repeat protein 7; RXRA, retinoid X receptor-alpha; FIBCD1, fibrinogen C domain containing 1; SALL4, Sal-like protein 4; KIF3C; kinesin family protein 3C; KDM5D, lysine (K)-specific demethylase 5D; FBXW8, F-box/WD repeat-containing protein 8; MUC17, myotubularin related protein; CCR5, chemokine (C-C motif) receptor 5; PDK2, pyruvate dehydrogenase kinase isoform 2; GSTM4, glutathione S-transferase Mu 4; DHRS2, dehydrogenase/reductase member 2; SRSF5, serine/arginine-rich splicing factor 5; HGS, hepatocyte growth factor-regulated tyrosine kinase substrate; TRIL, TLR4 interact with leucine-rich repeats.
(DOC)

S3 Table List of primers used for cloning in this study. The sequences of primer involved in this study are list in Table. F, forward; R, reverse. The letters in italics indicate as restriction enzyme sites.
(DOC)
