# Supplementary figures and images for: HRS plays an important role for TLR7 signaling to orchestrate inflammation and innate immunity upon EV71 infection
Source: PLoS Pathog. 2017 Aug 30;13(8):e1006585. doi: 10.1371/journal.ppat.1006585 (PMC5595348; doi:10.1371/journal.ppat.1006585)

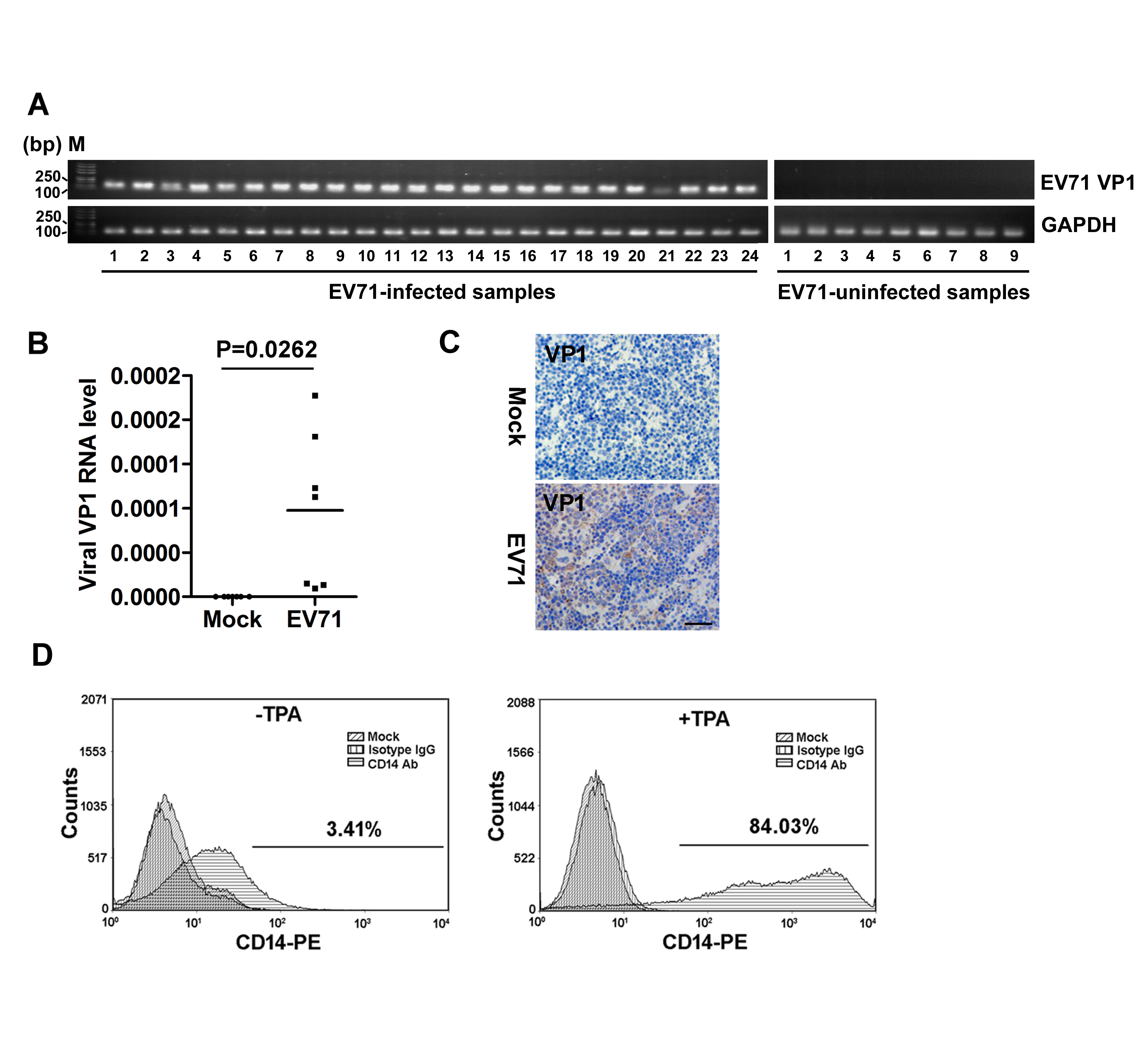

Supplement: S1 Fig — (A) PMBCs were isolated from EV71-infected or uninfected persons. Total RNA was extracted, followed by semi-RT PCR with specific EV71 VP1 and GAPDH (an internal control) primers. The results represent the EV71 RNA detection in all collected samples. (B and C) EV71 VP1 viral RNA (B) and protein (C) expressed in PBMCs and spleen of EV71-infected or non-infected (Mock) mice (each group, n = 7) were measured using qPCR and IHC, respectively. (D) Macrophages were differentiating from human THP-1 cells by the treatment of 100 nM TPA. CD14 was selected as a cell surface marker of macrophage derived from THP-1 cells. The surface marker of differentiated macrophages was detected by flow cytometry. Histograms of median fluorescent intensity of CD14 of THP-1 cells untreated (-TPA) or treated with TPA (+TPA). Data are representative of three independent experiments. (TIF) [file ppat.1006585.s001.tif]

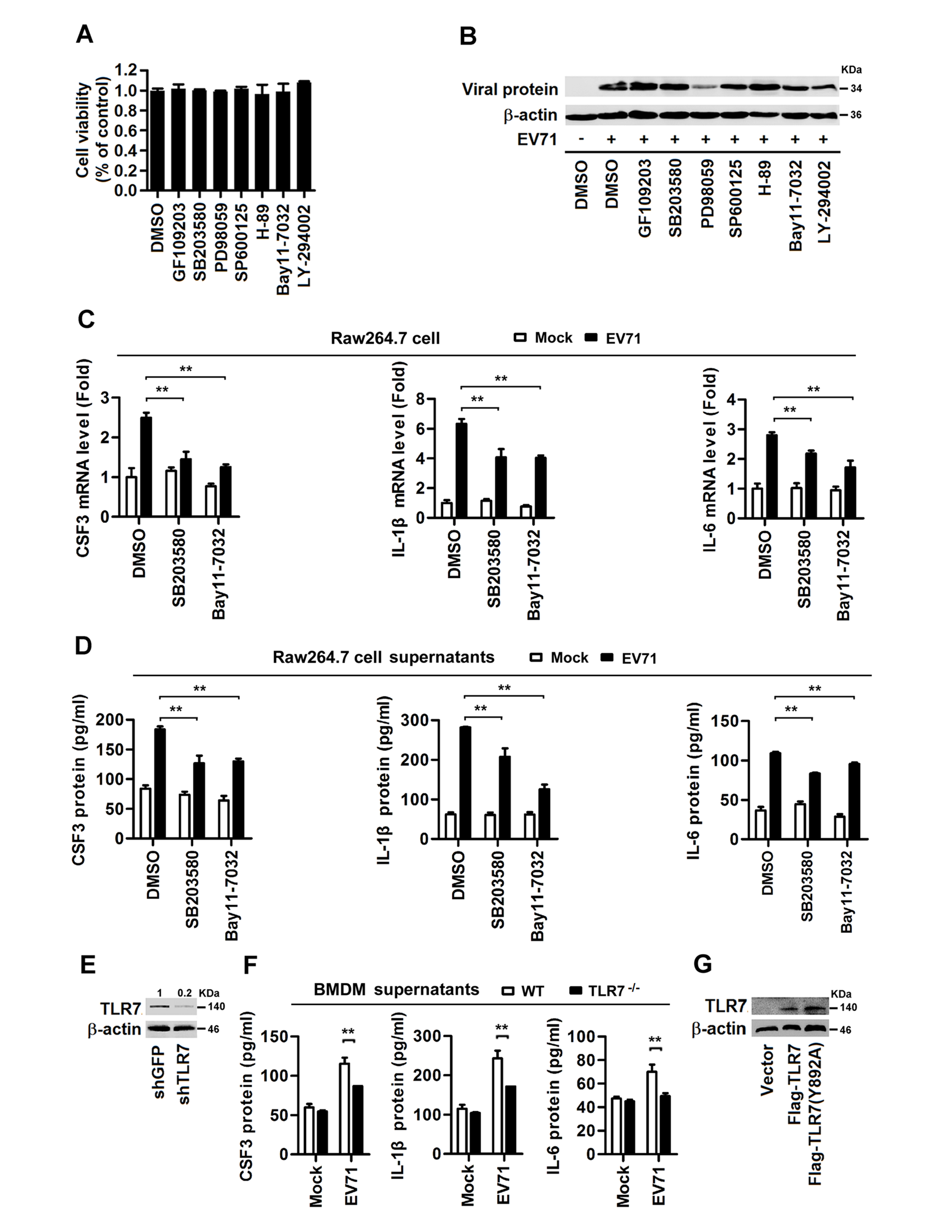

Supplement: S2 Fig — (A) THP-1 cells were treated with individual kinase inhibitors for 6 h, as indicated. Cell viabilities were then determined. (B) THP-1 cells were treated with individual kinase inhibitors for 6 h, as indicated, and infected with EV71 for 12 h. EV71 3C and β-actin proteins were detected by Western blotting analyses. (C and D) Mouse Raw264.7 cells were treated with indicated kinase inhibitors for 6 h and infected with EV71 (MOI = 5) for 24 h. CSF3, IL-1β, and IL-6 mRNAs (C) and supernatants CSF3, IL-1β, and IL-6 proteins (D) were measured by qPCR and ELISA, respectively. (E) THP-1 cells were transfected with shTLR7 or shGFP, and selected with 300 μg/ml G418. TLR7 and β-actin proteins expressed in the cells were detected by Western blotting analyses using specific antibodies to the proteins. (F) Mouse bone marrow-derived macrophages (BMDM) isolated from TLR7 wild-type (WT) or TLR7 knock-out (TLR7-/-) mice were infected with EV71 (MOI = 5) for 24 h. The mouse CSF3, IL-1β, and IL-6 proteins in cell supernatants were measured by ELISA. (G) HEK293T cells were transfected with pFlag-TLR7, pFlag-TLR7(Y892A) (a mutant of TLR7), or the vector. TLR7 and β-actin proteins expressed in the cells were detected by Western blotting analyses using specific antibodies to the proteins. Data are shown as mean ± SD and correspond to a representative experiment out of three performed. ns, non-significant; *, P < 0.05; **, P < 0.01. (TIF) [file ppat.1006585.s002.tif]

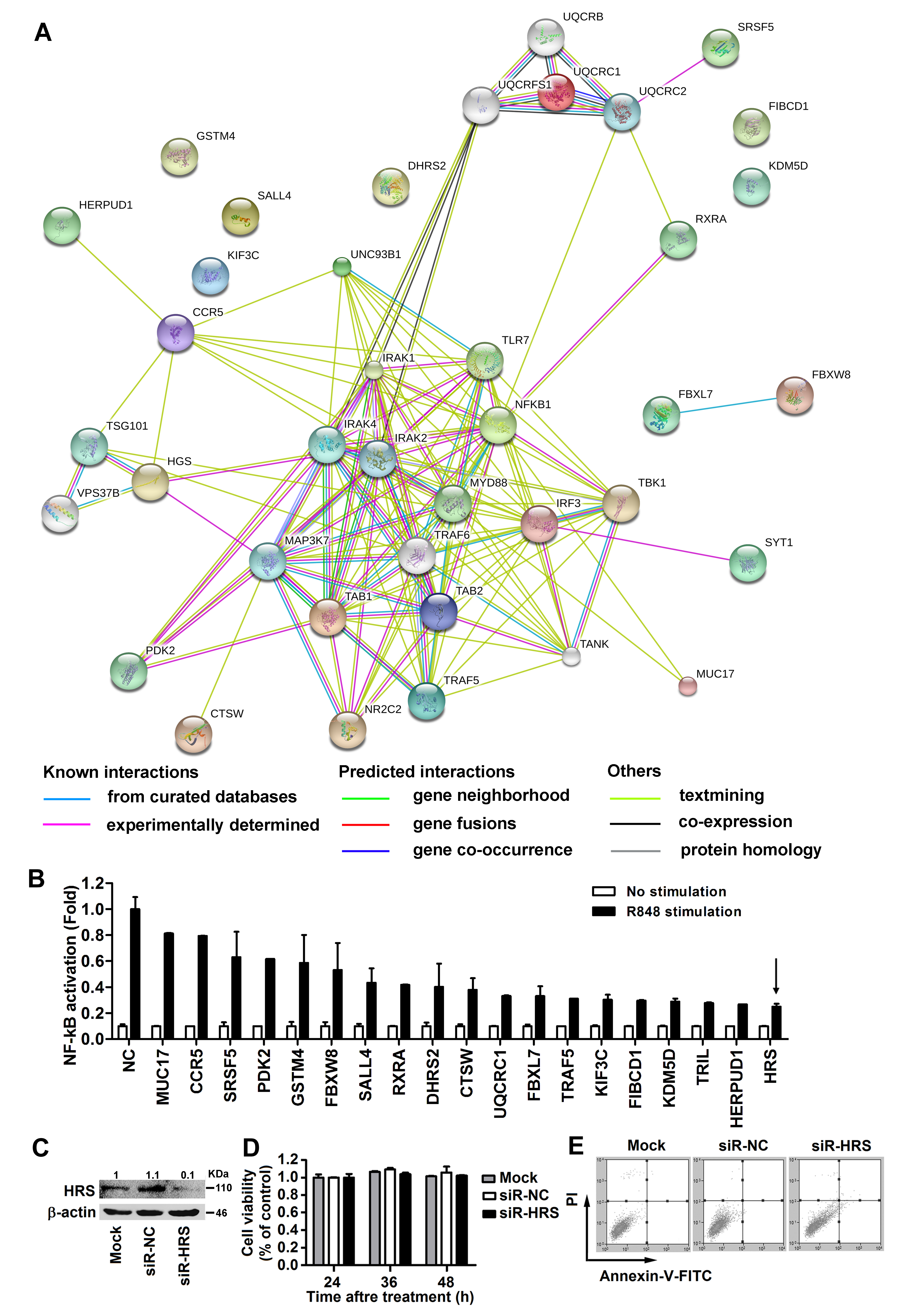

Supplement: S3 Fig — (A) Identified TLR7 signaling pathway associated factors and unknown or predicted proteins are integrated into available STRING database using version 10.0 of STRING software (http://string-db.org). Total selected 28 items represent in a form of node and the lines in different colors stand for the known or predicted interactions in TLR7 signaling pathway. (B) Stable HEK293T/TLR7/NF-κB reporter cells were transfected with plasmids encoding siRNAs specific to indicated genes and stimulated with R848. NF-κB activities were determined by luciferase activity assays. (C) THP-1 cells were transiently transfected with siRNA to HRS (siR-HRS) or its negative control (siR-NC) for 36 h. HRS and β-actin proteins were detected by Western blotting analyses. (D) THP-1 cells were transfected with siR-HRS or siR-NC for 24, 36, and 48 h. The cell viabilities were determined. (E) THP-1 cells were transfected with siR-HRS or siR-NC, and treated with Annexin V: FITC. The cell apoptosis was analyzed by Apoptosis Detection Kit (BD Biosciences, San Jose, CA). (TIF) [file ppat.1006585.s003.tif]

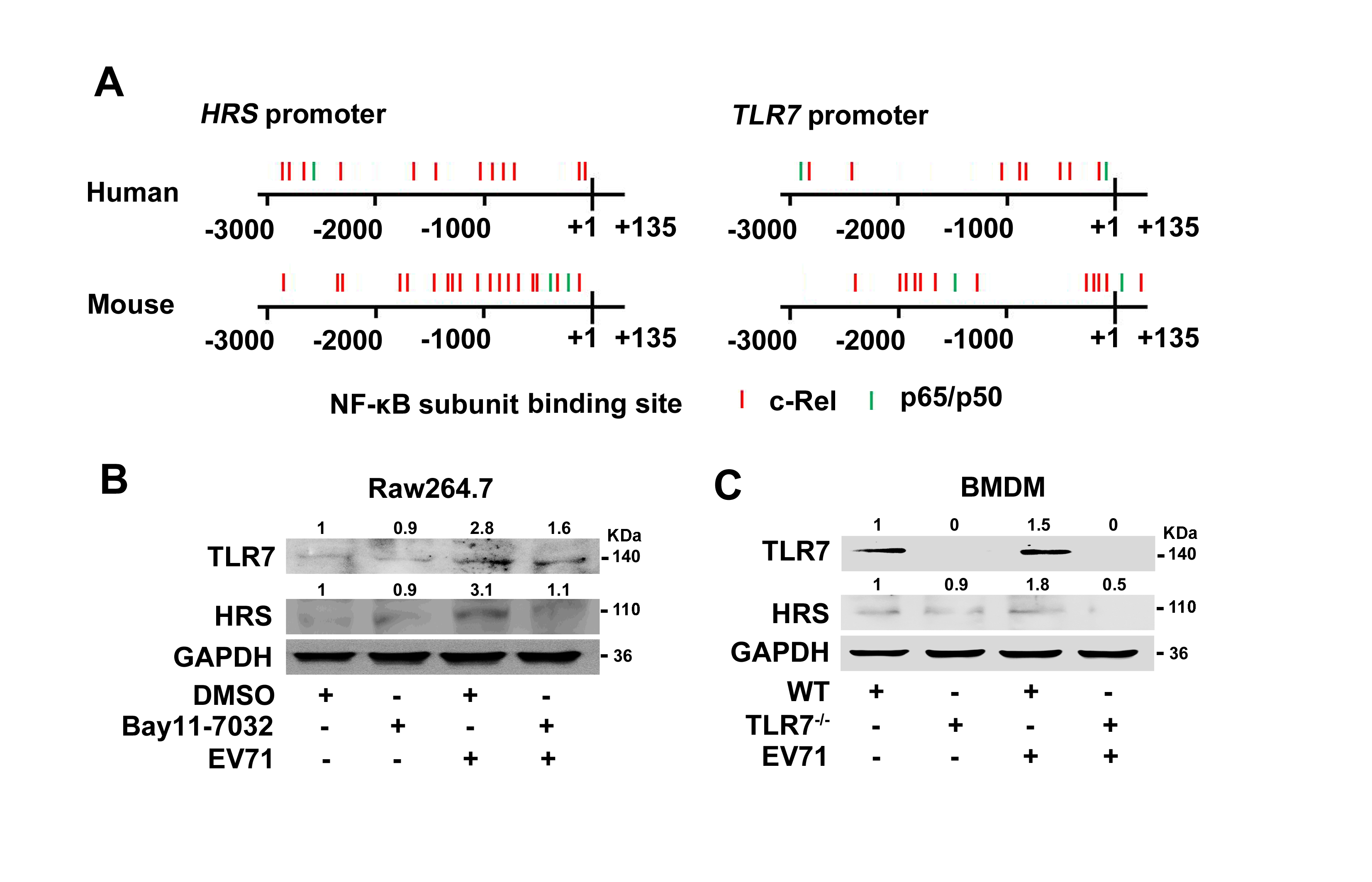

Supplement: S4 Fig — (A) Bioinformatic prediction of NF-κB subunit binding sites in human and mouse HRS or TLR7 promoter using P-Match 1.0 Public software (http://gene-regulation.com/). (B) Mouse Raw264.7 cells were treated with indicated kinase inhibitors for 6 h, and infected with EV71 (MOI = 5) for 24 h. (C) Mouse bone marrow-derived macrophages (BMDM) isolated from TLR7 WT or TLR7-/- mice were infected with EV71 (MOI = 5) for 24 h. (B and C) The proteins expressed in the treated cells were detected by Western blotting. The indicated band intensity represents as fold changes to internal control by using Image J software analysis. (TIF) [file ppat.1006585.s004.tif]

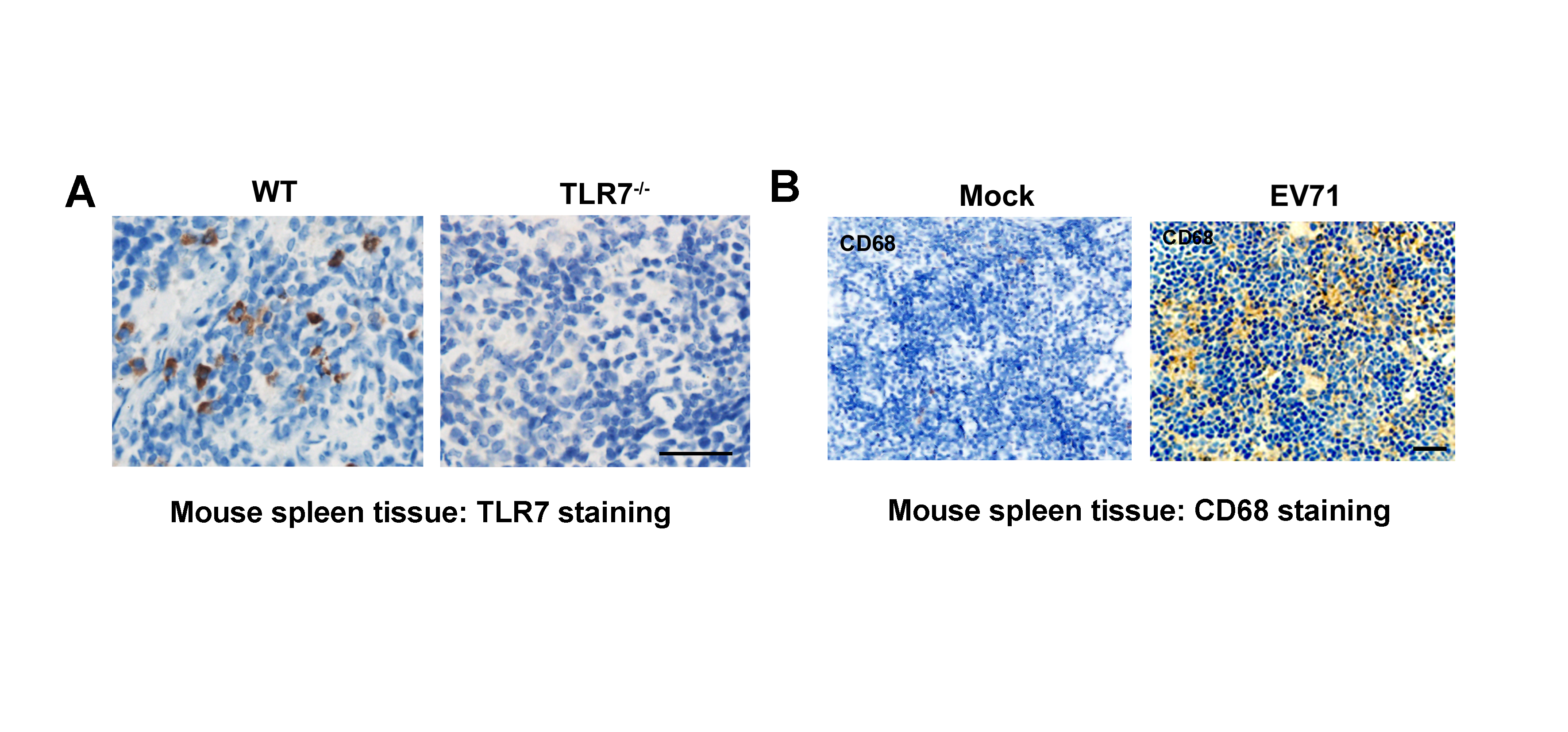

Supplement: S5 Fig — (A) Mice spleens from WT or TLR7-/- mice were subjected to immunohistochemistry (IHC) staining with TLR7 antibody. Bar = 100 μm. (B) Mice were mock-infected or infected with EV71 and sacrificed at indicated period. Mice spleens were subjected to immunohistochemistry (IHC) staining with the anti-mouse CD68 antibody. Bar = 50 μm. (TIF) [file ppat.1006585.s005.tif]

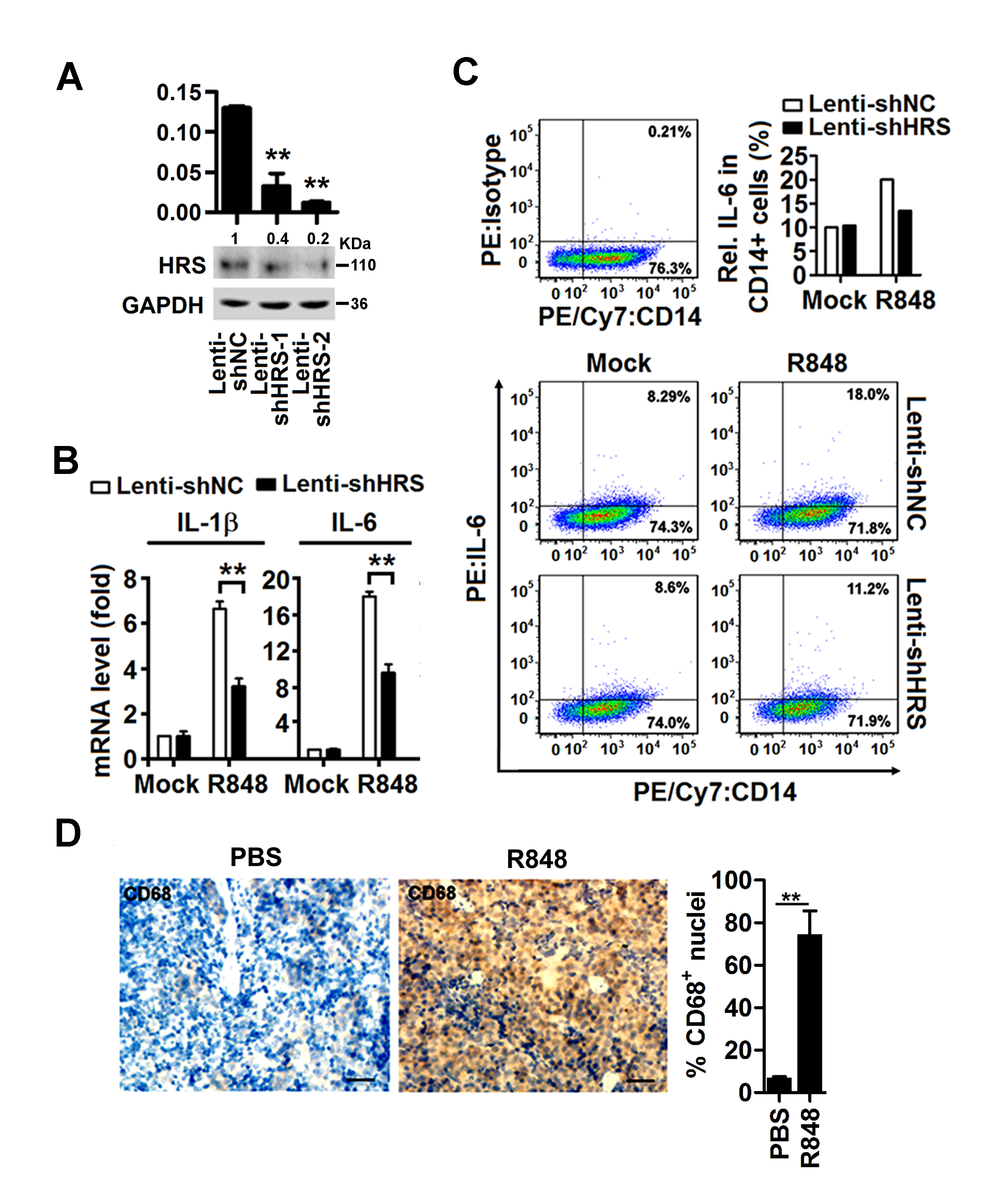

Supplement: S6 Fig — (A) Mouse Bone marrow-derived macrophages (BMDMs) isolated from mice were infected with lentivirus coding siRNA to HRS (Lenti-siR-HRS-1 and -2) or the control (Lenti-siR-NC) for 72 h. The efficiency of knock-down of HRS is evaluated by the determination of HRS mRNA and HRS protein using qPCR (upper panel) and Western blotting analyses (lower panel). (B and C) BMDMs isolated from mice were infected with lentivirus coding siRNA to HRS or the control for 72 h and stimulated with or without R848 (100 ng/ml) for 12 h. IL-1β and IL-6 mRNA levels were determined using qPCR (B). IL-6 protein levels were determined using flow cytometry (C). (D) Mice were stimulated without or with R848 and sacrificed at indicated period. Mice spleens were subjected to immunohistochemistry (IHC) staining with the anti-mouse CD68 antibody. Bar = 50 μm. Results were expressed as fold induction relative to control. **, P < 0.01. (TIF) [file ppat.1006585.s006.tif]

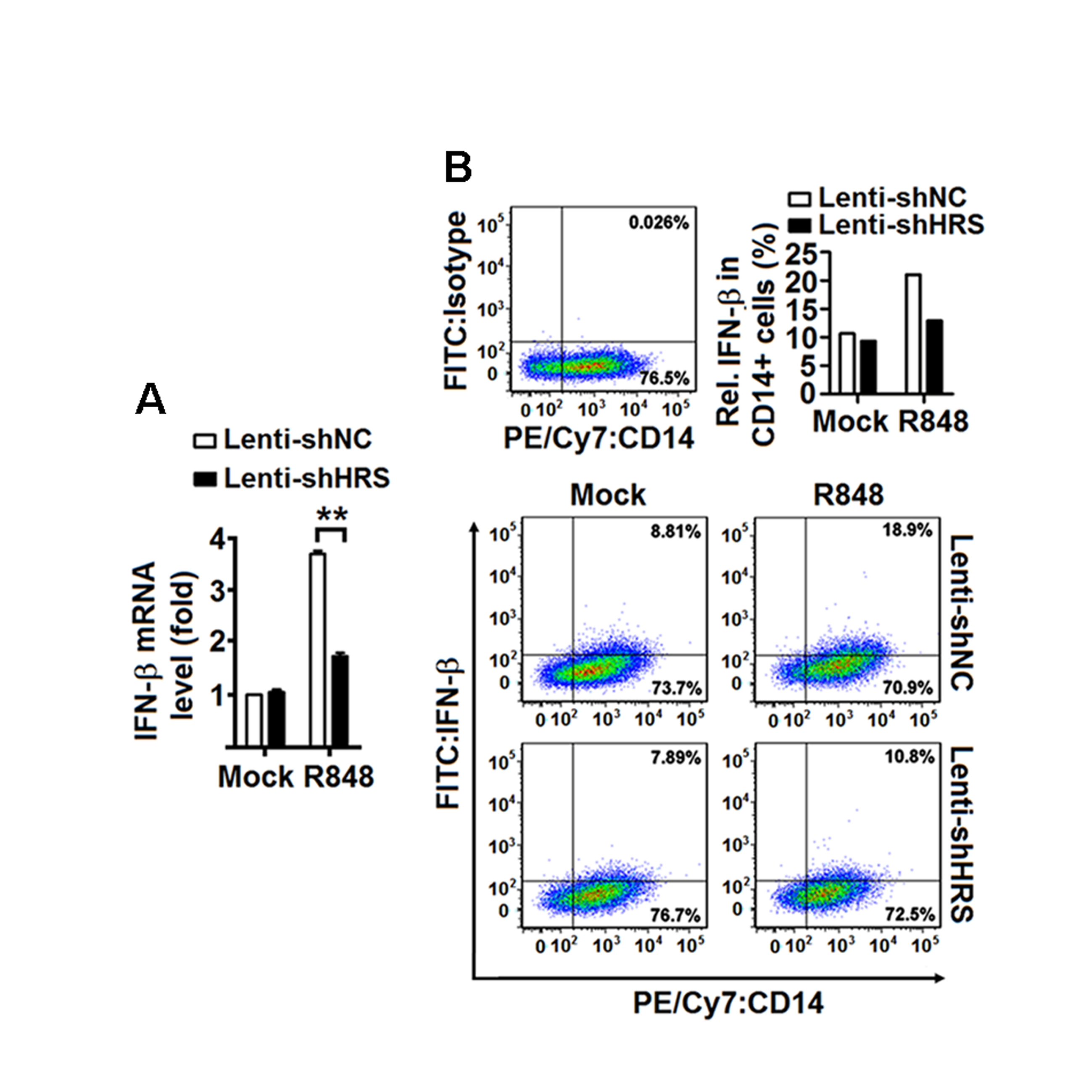

Supplement: S7 Fig — (A) Macrophages were transfected with siR-NC or siR-HRS and stimulated with R848. The level of IFN-β mRNA was determined using qPCR. (B) BMDMs isolated from mice were infected with lentivirus coding siRNA to HRS or the control for 72 h and stimulated with or without R848 (100 ng/ml) for 12 h. IFN-β protein level was determined using flow cytometry. (TIF) [file ppat.1006585.s007.tif]
