# Supplementary material for: HRS plays an important role for TLR7 signaling to orchestrate inflammation and innate immunity upon EV71 infection
Source: PLoS Pathog. 2017 Aug 30;13(8):e1006585. doi: 10.1371/journal.ppat.1006585 (PMC5595348; doi:10.1371/journal.ppat.1006585)
Supplement: S1 Table — THP-1 derived macrophages were infected with EV71 (at MOI = 5) or inoculated with UV-inactivated EV71 for 12 h. Levels of cytokines in cell supernatants were measured by a commercial kit (Human Cytokine ELISA Plate Assay I for Profiling 30 Cytokines kit, Signosis, Sunnyvale, CA, USA) according to manufacturers’ instructions. Data are shown as fold changes of protein expression in cell supernatants compared to mock samples. At least 2 times changes were considered statistically significant. *TNF-α, tumor necrosis factor alpha; IFN-γ, interferon gamma; CSF3, colony stimulating factor 3; GM-CSF, granulocyte-macrophage colony stimulating factor; IL-1α, interleukin 1 alpha; VEGF, vascular endothelial growth factor; EGF, epidermal growth factor; IL-6, interleukin 6; Resistin, also called RSTN or RETN1; PAI-1, plasminogen activator inhibitor type 1; IL-12, interleukin 12; IL-13, interleukin 13; Eotaxin-3, also named chemokine (C-C motif) ligand 26 (CCL26); PDGF-BB, platelet-derived growth factor beta polypeptide; PIGF-1, placental growth factor 1; β-NGF, nerve growth factor-beta; SCF, skp-cullin-F-box; MCP-1, monocyte chemotactic protein 1; MIP-1α, macrophage inflammatory protein 1 alpha; IL-2, interleukin 2; IL-4, interleukin 4; IL-8, interleukin 8; IL-10, interleukin 10; bFGF, basic fibroblast growth factor; Leptin, also called LEPD; IGF-1, insulin-like growth factor 1; TGF-β, transforming growth factor beta; Adipo, adiponectin; IL-17α, interleukin 17 alpha; IL-1β, interleukin 1 beta. (DOC) [file ppat.1006585.s008.doc]

| Human Cytokine ELISA Plate Array | | | | | | | |
| --- | --- | --- | --- | --- | --- | --- | --- |
| Item | | Intensity Value | | | Fold Change | | |
| No | Cytokine* | Mock | Inactive EV71 | EV71 | Mock | Inactive EV71 | EV71 |
| 1 | TNF-α | 10369 | 6041 | 9241 | 1 | 0.582601987 | 0.89121 |
| 2 | IFN-γ | 87630 | 73937 | 41756 | 1 | 0.843740728 | 0.4765 |
| 3 | CSF3 | 38897 | 50909 | 82755 | 1 | 1.30881559 | 2.12754 |
| 4 | GM-CSF | 90403 | 52951 | 133846 | 1 | 0.585721713 | 1.48055 |
| 5 | IL-1α | 166564 | 42400 | 101290 | 1 | 0.254556807 | 0.60811 |
| 6 | VEGF | 87961 | 76467 | 146052 | 1 | 0.869328452 | 1.66042 |
| 7 | EGF | 11070 | 5285 | 17839 | 1 | 0.477416441 | 1.61147 |
| 8 | IL-6 | 78846 | 74125 | 168536 | 1 | 0.940123786 | 2.13753 |
| 9 | Resistin | 147060 | 77898 | 117591 | 1 | 0.529702162 | 0.79961 |
| 10 | PAI-1 | 8196 | 4536 | 19408 | 1 | 0.553440703 | 2.36798 |
| 11 | IL-12 | 107020 | 83363 | 167958 | 1 | 0.77894786 | 1.56941 |
| 12 | IL-13 | 10007 | 8002 | 18310 | 1 | 0.799640252 | 1.82972 |
| 13 | Eotaxin-3 | 84985 | 50819 | 94096 | 1 | 0.597976113 | 1.10721 |
| 14 | PDGF-BB | 11401 | 7633 | 13040 | 1 | 0.669502675 | 1.14376 |
| 15 | PIGF-1 | 62952 | 38273 | 112504 | 1 | 0.607971153 | 1.78714 |
| 16 | β-NGF | 6336 | 6177 | 15075 | 1 | 0.974905303 | 2.37926 |
| 17 | SCF | 83963 | 88811 | 130433 | 1 | 1.057739719 | 1.55346 |
| 18 | MCP-1 | 99001 | 47211 | 125049 | 1 | 0.476873971 | 1.26311 |
| 19 | MIP-1α | 75578 | 91793 | 107670 | 1 | 1.214546561 | 1.42462 |
| 20 | IL-2 | 101428 | 71919 | 124576 | 1 | 0.709064558 | 1.22822 |
| 21 | IL-4 | 64020 | 44843 | 123782 | 1 | 0.700452983 | 1.93349 |
| 22 | IL-8 | 42473 | 38963 | 76811 | 1 | 0.917359264 | 1.80847 |
| 23 | IL-10 | 8567 | 9314 | 34834 | 1 | 1.087195051 | 4.06607 |
| 24 | bFGF | 5316 | 8395 | 25147 | 1 | 1.579194883 | 4.73044 |
| 25 | Leptin | 2976 | 4115 | 9257 | 1 | 1.382728495 | 3.11055 |
| 26 | IGF-1 | 75172 | 80804 | 190890 | 1 | 1.074921513 | 2.53938 |
| 27 | TGF-β | 8436 | 9206 | 23244 | 1 | 1.091275486 | 2.75533 |
| 28 | Adipo | 5486 | 5279 | 6586 | 1 | 0.96226759 | 1.20051 |
| 29 | IL-17α | 6290 | 8683 | 21131 | 1 | 1.380445151 | 3.35946 |
| 30 | IL-1β | 11497 | 11134 | 30066 | 1 | 0.968426546 | 2.61512 |
