# Supplementary material for: HRS plays an important role for TLR7 signaling to orchestrate inflammation and innate immunity upon EV71 infection
Source: PLoS Pathog. 2017 Aug 30;13(8):e1006585. doi: 10.1371/journal.ppat.1006585 (PMC5595348; doi:10.1371/journal.ppat.1006585)
Supplement: S2 Table — UQCRC1, ubiquinol-cytochrome C reductase core protein I; CTSW, cathepsin W; TRAF5, tumor necrosis factor receptor-associated factor 5; HERPUD1, homocysteine-inducible, endoplasmic reticulum stress-inducible, ubiquitin-like domain member 1; FBXL7, F-box and leucine-rich repeat protein 7; RXRA, retinoid X receptor-alpha; FIBCD1, fibrinogen C domain containing 1; SALL4, Sal-like protein 4; KIF3C; kinesin family protein 3C; KDM5D, lysine (K)-specific demethylase 5D; FBXW8, F-box/WD repeat-containing protein 8; MUC17, myotubularin related protein; CCR5, chemokine (C-C motif) receptor 5; PDK2, pyruvate dehydrogenase kinase isoform 2; GSTM4, glutathione S-transferase Mu 4; DHRS2, dehydrogenase/reductase member 2; SRSF5, serine/arginine-rich splicing factor 5; HGS, hepatocyte growth factor-regulated tyrosine kinase substrate; TRIL, TLR4 interact with leucine-rich repeats. (DOC) [file ppat.1006585.s009.doc]

| No. | Gene Symbol | Genbank Accession | Gene Function Description |
| --- | --- | --- | --- |
| 1 | UQCRC1 | NM_003365 | a mitochondrial precursor |
| 2 | CTSW | NM_001335 | a cysteine proteinnase, associated with inside of the ER |
| 3 | TRAF5 | NM_004619 | mediating TNF receptor signal transduction |
| 4 | HERPUD1 | NM_014685 | located in ER for ubiquitination |
| 5 | FBXL7 | NM_012304 | mediator for phosphorylation-dependent ubiquitination proteins |
| 6 | RXRA | NM_002957 | a steroid and thyroid hormone transcriptional regulators. |
| 7 | FIBCD1 | NM_032843 | a transmembrane endocytic receptor |
| 8 | SALL4 | NM_020436 | a zinc finger transcription factor |
| 9 | KIF3C | NM_002254 | kinesin family member |
| 10 | KDM5D | NM_004653 | a zinc finger transcription factor |
| 11 | FBXW8 | NM_153348 | a member of the F-box protein family, function in phosphorylation-dependent ubiquitination |
| 12 | MUC17 | NM_001040105 | membrane mucins |
| 13 | CCR5 | NM_000579 | a member of the beta chemokine receptor family |
| 14 | PDK2 | NM_002611 | a member of pyruvate dehydrogenase kinase family |
| 15 | GSTM4 | NM_147148 | mu class of enzymes functions in the detoxification |
| 16 | DHRS2 | NM_182908 | a member of dehydrogenase/reductase family |
| 17 | SRSF5 | NM_001039465 | serine/arginine-rich family of pre-mRNA splicing factor |
| 18 | HGS | NM_004712 | an endosomal regulator |
| 19 | TRIL | NM_014817 | a component of the TLR4 complex |
