# Supplementary material for: HRS plays an important role for TLR7 signaling to orchestrate inflammation and innate immunity upon EV71 infection
Source: PLoS Pathog. 2017 Aug 30;13(8):e1006585. doi: 10.1371/journal.ppat.1006585 (PMC5595348; doi:10.1371/journal.ppat.1006585)
Supplement: S3 Table — The sequences of primer involved in this study are list in Table. F, forward; R, reverse. The letters in italics indicate as restriction enzyme sites. (DOC) [file ppat.1006585.s010.doc]

| Primer title | Orientation |
| --- | --- |
| TLR7 F (*Bam*HI) | 5’-TAA*GGATCC*ATGGTGTTTCCAATGTGGACA |
| TLR7 R (*Sal*I) | 5’-TTA*GTCGAC*CTAGACCGTTTCCTTGAACACCT |
| HRS F (*Xho*I) | 5’-AAT*CTCGAG*ATGGGGCGAGGCAGCGGCACCTT |
| HRS R (*Kpn*I) | 5’-CTT*GGTACC*GATCTGTCGAATGAAATGAGCT |
| dFYVE F | 5’-GTTCAGCTGCTCGTATTCCTCAGCGTCCAC |
| dFYVE R | 5’-GTTCAGCTGCTCGTATTCCTCAGCGTCCAC |
| TLR7 (VN-173) F | 5’-AAAGACGATGACGAC*AAGCTTA*TGGTGTTTCCAATGTGGACA |
| TLR7 (VN-173) R | 5’-GATGGATCTTCTAGA*GTCGAC*GACCGTTTCCTTGAACACCT |
| TAB1 (VN-173) F | 5’-AAAGACGATGACGAC*AAGCTT*ATGGCGGCGCAGAGGA |
| TAB1 (VN-173) R | 5’-GATGGATCTTCTAGA*GTCGAC*CCCTGGGGTCAGGCT |
| HRS (VC-155) F | 5’-TGGCCATGGAGGCCC*GAATTC*GGATGGGGCGAGGCAGCGGCACCTT |
| HRS (VC-155) R | 5’-TTTGCACGCCGGACG*GGTACC*GTCGAATGAAATGAGCT |
